# Easy, Robust, and Repeatable Online Acid Cleavage of Proteins in Mobile Phase for Fast Quantitative LC-MS Bottom-Up Protein Analysis—Application for Ricin Detection

**DOI:** 10.1021/acs.analchem.3c01772

**Published:** 2023-08-11

**Authors:** Denis K. Naplekov, Siddharth Jadeja, Alena Myslivcová Fučíková, František Švec, Hana Sklenářová, Juraj Lenčo

**Affiliations:** † Department of Analytical Chemistry, Faculty of Pharmacy in Hradec Králové, Charles University, Heyrovského 1203/8, 500 05 Hradec Králové, Czech Republic; ‡ Department of Biology, Faculty of Science, University of Hradec Králové, Hradecká 1285, 500 03 Hradec Králové, Czech Republic

## Abstract

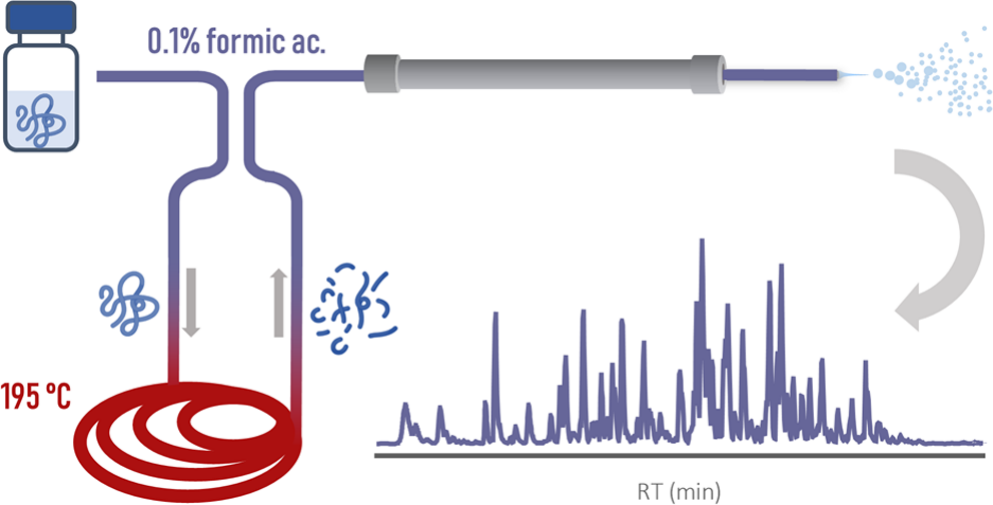

Sample preparation involving the cleavage of proteins into peptides is the first critical step for successful bottom-up proteomics and protein analyses. Time- and labor-intensiveness are among the bottlenecks of the commonly used methods for protein sample preparation. Here, we report a fast online method for postinjection acid cleavage of proteins directly in the mobile phase typically used for LC-MS analyses in proteomics. The chemical cleavage is achieved in 0.1% formic acid within 35 s in a capillary heated to 195 °C installed upstream of the analytical column, enabling the generated peptides to be separated. The peptides generated by the optimized method covered the entire sequence except for one amino acid of trastuzumab used for the method development. The qualitative results are extraordinarily stable, even over a long period of time. Moreover, the method is also suitable for accurate and repeatable quantification. The procedure requires only one manual step, significantly decreasing sample transfer losses. To demonstrate its practical utility, we tested the method for the fast detection of ricin. Ricin can be unambiguously identified from an injection of 10 ng, and the results can be obtained within 7–8 min after receiving a suspicious sample. Because no sophisticated accessories and no additional reagents are needed, the method can be seamlessly transferred to any laboratory for high-throughput proteomic workflows.

Protein cleavage into peptides is a crucial step in a bottom-up approach that has represented the mainstream in proteomics and protein analyses for around two decades. Trypsin is the preferred protease to generate proteome-representative peptides since they possess properties well compatible with the subsequent LC-MS analysis.^1^ The standard benchtop protein digestion is time-consuming.^2−4^ Various attempts have been made to accelerate the digestion,^3^ but the optimized workflows remain labor-intensive. Methods relying on immobilized protease for online integration of the digestion to LC-MS have also been developed.^3,5−7^ Obviously, except for proteases favoring low pH, such as pepsin, these can not be carried out using simple LC instruments to ensure that the digestion buffer and the mobile phase for the separation of peptides have optimum composition. Moreover, all reagents in protein samples must be compatible with the enzyme activity. Ultrafast enzymatic digestion methods that can be online coupled with MS analysis have also been very recently introduced.^8,9^ However, these methods that require sophisticated and in-house manufactured accessories do not allow the separation of generated peptides nor their clean-up. Easy, fast, and robust conversion of proteins in peptides could thus become a bottleneck for the recently emerged fast and ultrafast LC-MS proteomic strategies.^10−14^ More importantly, specific applications exist where not only the time but also the extent of sample handling for its preparation is an important aspect, such as the detection of protein toxins.

We recently noticed that peptides and proteins could readily hydrolyze in the mobile phase for reversed-phase liquid chromatography acidified using 0.1% formic acid at higher column temperatures.^15,16^ The rapid, yet not complete, hydrolysis allowed us to identify 13 peptides when reduced trastuzumab was chromatographed at 90 °C using a 14 min gradient.^15^ The observed specificity toward Asp indicated that the chemical hydrolysis was due to low pH. The remarkable productivity of the in-column acid hydrolysis directly in the mobile phase inspired us to attempt adopting it for fast, automated, yet not too instrumentally complex chemical cleavage of proteins seamlessly integrable in LC-MS bottom-up analyses.

The acid cleavage of peptide bonds has been attracting the attention of researchers since the 1950s when Asp was identified as the fastest amino acid released from proteins incubated at high temperatures.^17−21^ The supposed specificity of the acid cleavage was later adopted by Li et al. for protein identification using MS.^22^ It took only 2 h and was thus dramatically faster than the standard overnight trypsin digestion. To fully exploit this advantage for high-throughput proteomic workflows, attempts have been undertaken to accelerate the method using microwave radiation^23−28^ and increasing the temperature.^29−31^ Rapid cleavage of proteins preferred at Asp residue was also described in subcritical water.^32^ Yet, no offline method based on acid cleavage of proteins has gained wide popularity in proteomics, partly also because the time savings were insufficient and/or the workflows remained as labor-intensive as in standard trypsin digestion. Moreover, no method truly allowed its simple online integration to LC-MS with the possibility of separating the cleavage products because sophisticated accessories not broadly available to other researchers were required. Besides, the quantification performance of the acid cleavage has never been examined. We trust that the applicability of the acid cleavage in protein analysis can considerably increase by developing a truly simple, effective, and quantitative method seamlessly integrable into LC-MS. Such a method can also be particularly valuable for the fast detection of protein toxins, such as ricin.

Ricin produced in the castor bean seeds (*Ricinus communis*, Euphorbiaceae) is a heterodimer composed of two chains. Chain B is a lectin that mediates the internalization of ricin. Chain A is released upon reducing the interchain disulfide bond in the endoplasmic reticulum, and subsequently, it irreversibly deadenylates the catalytic 28S rRNA of ribosomes. The mechanism of action makes ricin one of the most lethal substances known.^33−36^ Because of its toxicity, relative availability, ease of production, and the absence of effective antidotes for treating ricin poisoning, the Centers for Disease Control and Prevention categorizes it as a tier B bioterrorism agent, and it is listed in the Chemical Weapons Convention under Schedule 1 compounds. Various analytical technologies have been applied to sensitive ricin detection to monitor its potential misuse, including the bottom-up approach.^37−41^ However, existing LC-MS-based proteomic methods cannot provide results within a few or several minutes after receiving a sample and typically involve extensive handling of potentially hazardous material.^42,43^

Inspired by our previous results, we elaborated in this study on the concept of fast online protein cleavage directly in the acidified mobile phase. We constructed a simple, inexpensive apparatus, fully integrable online to LC-MS systems, that enabled protein cleavage within 35 s. Upon optimization, the method demonstrated outstanding qualitative repeatability, quantitative reliability, and overall robustness. Subsequently, as a proof of concept, we demonstrate its application potential for the fast detection of ricin involving only a single manual step.

## Experimental

### Reagents and Materials

Unless otherwise stated, all chemicals and reagents were purchased from Sigma-Aldrich/Merck in the highest available grade. LC-MS-grade solvents and additives to mobile phases were from Merck and Honeywell. Expired leftovers of reconstituted trastuzumab (Herceptin, Roche) were received from Multiscan Pharma, Czech Republic.

### Sample Preparation

#### Reduction of Trastuzumab

Trastuzumab was used for the method development and its characterization. 20 μL of the reconstituted trastuzumab (21 μg/μL) was mixed with 60 μL of 8 M guanidine hydrochloride, incubated at 60 °C for 5 min, and subsequently reduced in 20 mM dithiothreitol at 60 °C for 30 min. Water was added to obtain a concentration of 1 μg/μL. The injection volume was 2 μL.

#### Ricin

The laboratory where the study was conducted is not authorized to manipulate with intact ricin. Ricin toxicity is minimized when the disulfide bond between chains A and B is reduced.^44^ Therefore, we mixed standards for ricin chains A (Sigma-Aldrich/Merck) and B (Vector Laboratories) in an equimolar ratio in an HPLC vial containing 1–2 mg of tris(2-carboxyethyl)phosphine (TCEP) to create a safe, chemically equivalent analyte. The material that came into contact with the sample was decontaminated, and the leftovers liquidated in sodium hypochlorite.^34^

### Apparatus for the Online Acid Cleavage of Proteins in the Mobile Phase

The Antoine equation^45^ with coefficients valid above the boiling points retrieved from the Dortmund Data Bank^46^ predicted that pressure above 15.5 bar is sufficient to keep them liquid at 200 °C. This limit was significantly less than the pressures that were predicted to generate by Kozeny–Carman equation and Poisulle law when 100% acetonitrile would percolate the column maintained at 80 °C (32 bar) and a 350 mm × 50 μm restriction capillary (40 bar) at a flow rate of 300 μL/min.^47,48^

**Figure 1:**
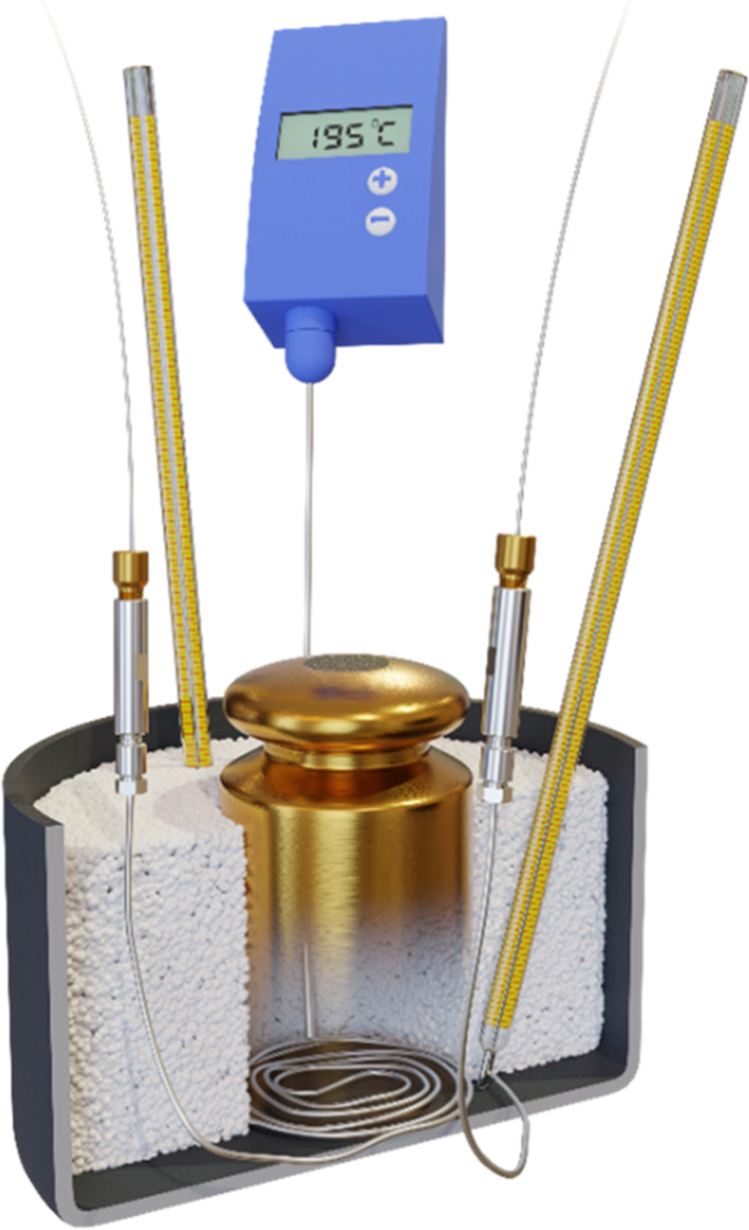
Simple apparatus used for the online acid cleavage of proteins in the mobile phase. The final configuration is shown, i.e., without the gradient mixer and with the alumina beads.

We intended to use only inexpensive LC-MS accessories and widely available laboratory equipment to make the method broadly transferrable. A 100 cm stainless steel capillary with an inner diameter of 0.020 in. (∼0.5 mm) was coiled to form a flat snail-like loop, with its middle in the center. The coiled reaction capillary was placed at the bottom of a laboratory pot and weighted down with a 2 kg cylindrical weight (Figures 1 and S1). A 10 μL gradient mixer was installed at the inlet end of the reaction capillary. The reaction capillary was connected to a two-position six-port valve that allowed analyzing samples also in their uncleaved form (Figure S2).

The space in the laboratory pot was filled with extra pure laboratory sand (Fisher Scientific). A hot plate stirrer IKA RET basic equipped with temperature control via a PT 1000.60 digital sensor (IKA-Werke, Germany) was used for heating. The probe tip was placed at the pot bottom along the wall of the 2 kg weight. The temperature on the digital sensor was allowed to fluctuate ±1 °C around the set temperature. Besides, two thermometers were placed at the pot bottom. The temperature they measured was allowed to fluctuate ±2 °C around the set temperature, but the average value of all three readouts had to be within ±1 °C from the set value.

### LC-MS Analyses

LC-MS analyses were carried out using a Vanquish Horizon UHPLC system hyphenated to a Q Exactive HF-X mass spectrometer (Thermo Fisher Scientific). Component A of the mobile phase was aqueous 0.1% formic acid, and component B was 0.1% formic acid in acetonitrile. The analytes were separated at 80 °C in a 2.1 × 100 mm BioResolve RP mAb column packed with 2.7 μm/450 Å superficially porous polyphenyl-bonded particles (Waters) at a flow rate of 300 μL/min using a gradient from 1 to 51% component B in 10 min that started after 1 min isocratic step. The first 2.35 min of the flow was diverted to waste. Eluted analytes were introduced into the mass spectrometer at 3.5 kV. MS1 spectra were recorded at 60,000 resolution within *m*/*z* 325–2000 with 1 × 10^6^ target ions and a maximum ion time of 60 ms. Three precursors with ≥2 and ≤5 charges were collisionally dissociated after reaching an intensity of 2.5 × 10^5^ using an apex-trigger option with an exclusion time of 3 s. An isolation window of 2.5 *m*/*z* and a normalized collision energy of 27 were used. MS2 spectra were acquired at a resolution of 30,000 with a 2 × 10^5^ AGC target and a maximum ion time of 150 ms. LC-MS files were deposited in the ProteomeXchange repository with the identifier PXD041124.

### LC-MS Data Evaluation

The LC-MS data were searched using Byonic v3.5 (Protein Metrics) against the FASTA sequence of trastuzumab and ricin. A nonspecific cleavage was chosen, and the mass tolerance was set at 5.5 ppm for precursors and 15 ppm for fragments. Trastuzumab and ricin peptides with NX[S/T] motif were screened for 57 N-glycans typical for human plasma proteins and 52 most common N-glycans in plants, respectively. Oxidized Met, dehydration of Asp, and pyroglutamate formation from N-terminal Glu and Gln were set as dynamic modifications. Because these modifications are artifacts linked to high temperature and low pH of the mobile phase,^15^ we report unique peptide sequences (uPSs) in this study, i.e., unique sequences regardless of the presence of the artificial modifications. For some tests, we also report peptides, i.e., peptide-spectrum matches discounting duplicates. Only spectra identified with a Byonic score of at least 300 were considered.^49^

For quantitative evaluation, spectra identification followed by spectral library building and MS1 peak extraction was performed in Skyline v22.2.^50^ Spectra were searched using the implemented MS Amanda.^51^ The nonspecific cleavage is not available in Skyline. Hence, a semispecific cleavage at both sites of Asp with a maximum of two missed cleavages was leveraged. The mass tolerance was set to 8 ppm for precursors and 18 ppm for fragments. Only pyroglutamate formation from N-terminal Gln was set as a dynamic modification. For peptides identified with a cut-off score of 0.99, Skyline extracted chromatograms for three monoisotopic peaks.

The dependency of peak area against the concentration of trastuzumab and ricin was examined in GraphPad v9.4 (GraphPad Software) from the total peak area of the monoisotopic peaks. GraphPad was also used for statistical evaluation. The quantitative Venn diagrams were prepared using BioVenn and redrawn in GraphPad.^52^ Unless otherwise stated, data were obtained in triplicates, and the error bars in the graphs represent the standard deviation. The second replicate was used for a representative demonstration.

## Results and Discussion

### Method Development and Optimization

#### Column

The efficiency of the acid cleavage was not known a priori, and we anticipated that large protein fragments might enter the column, thus representing a risk of its clogging (Note S1). Hence, we first selected a column that could simultaneously cope with the chromatographic properties of proteins and peptides.^48^ The BioResolve RP mAb column was designed for the chromatography of antibody biopharmaceuticals, their chains, and subunits.^53^ Besides, we recently revealed that the column is remarkably efficient also for separating peptides.^15,54^ The long polypeptides detected even under optimized conditions underlined the need to use a column that efficiently separates peptides of a size common for bottom-up proteomics but also larger protein fragments without any risk of column damage.

#### Temperature of the Reaction Capillary

Because of its open design, the temperature of the hot plate used to heat the capillary was limited to 200 °C for safety reasons. However, this temperature was not achievable due to air conditioning in the lab. Therefore, 195 °C was the highest temperature used in this study (197 °C was needed to be set). Temperatures ranging from 155 to 195 °C ramped in 10 °C increments were tested.

**Figure 2:**
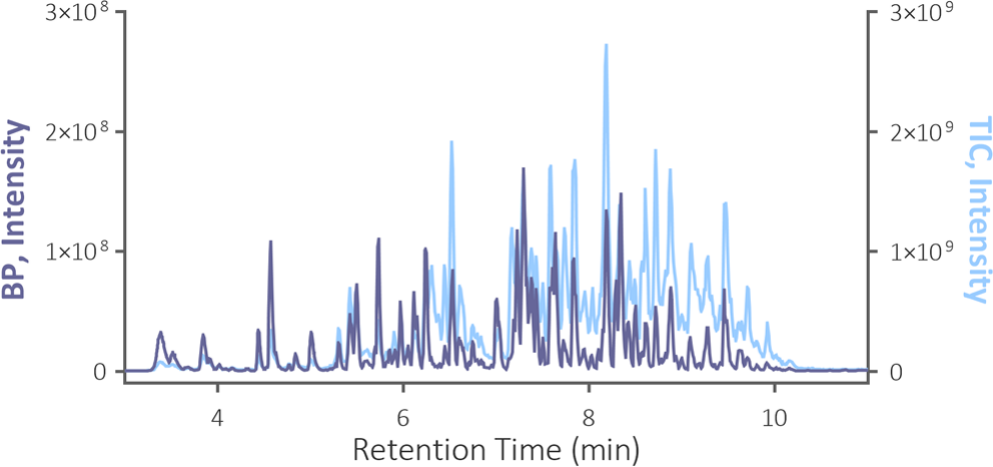
Base peak (BP) and total ion current (TIC) chromatograms acquired from trastuzumab cleaved in the reaction capillary at 195 °C.

A temperature of 155 °C already provided a promising cleavage efficiency (Figure S3). A substantial difference between the total ion current (TIC) and base peak (BP) chromatograms was registered at longer retention times, indicating the presence of long fragments with multiple charge states. Indeed, fragments with a deconvolved molecular weight of, for instance, 15,989, 17,922, and 18,278 Da were found in the MS spectra. The difference between the TIC and BP chromatograms and the abundance of highly charged precursors gradually decreased along with the rising temperature of the reaction capillary. Also, more peaks were detected at shorter retention times (Figure 2). These observations indicated more efficient cleavage generating smaller peptides from the large trastuzumab fragments eluted at longer retention times.

**Figure 3:**
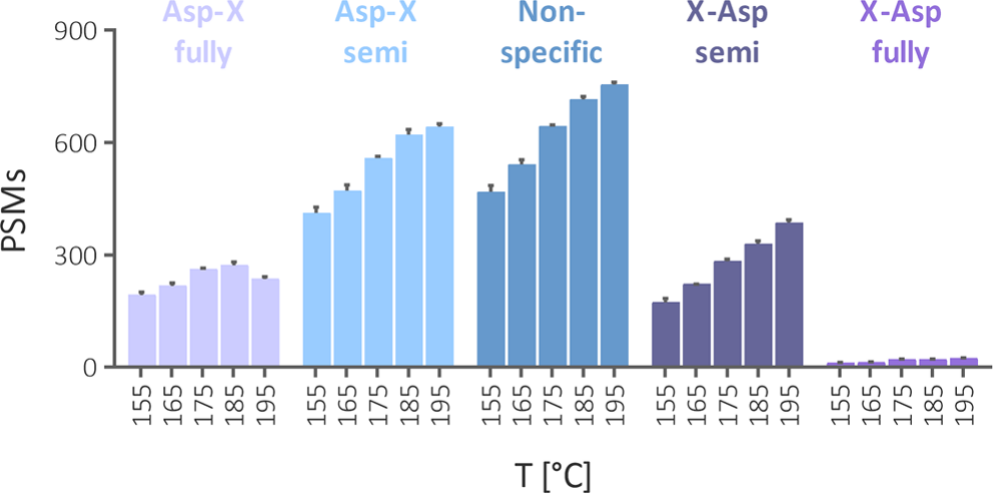
Effect of cleavage settings in Byonic search on the number of peptide-spectrum matches (PSM) at different temperatures.

The acid hydrolysis of proteins has been deemed to cleave Asp-X bonds selectively.^19−22^ However, in our settings, only a minor portion of MS2 spectra was identified using a specific search toward Asp-X cleavage with two missed cleavage sites (Figure 3). The number of identified spectra increased more than 2-fold when we applied a semispecific search and further by approximately 15% when we allowed a nonspecific cleavage. A specific search toward X-Asp cleavage identified minimum spectra, confirming a slower cleavage rate at the N-terminal site of Asp.^15,22^ Because of its superior performance and the opportunity to assess the cleavage specificity unbiasedly, we leveraged the nonspecific setting for qualitative analyses in this study.

**Figure 4:**
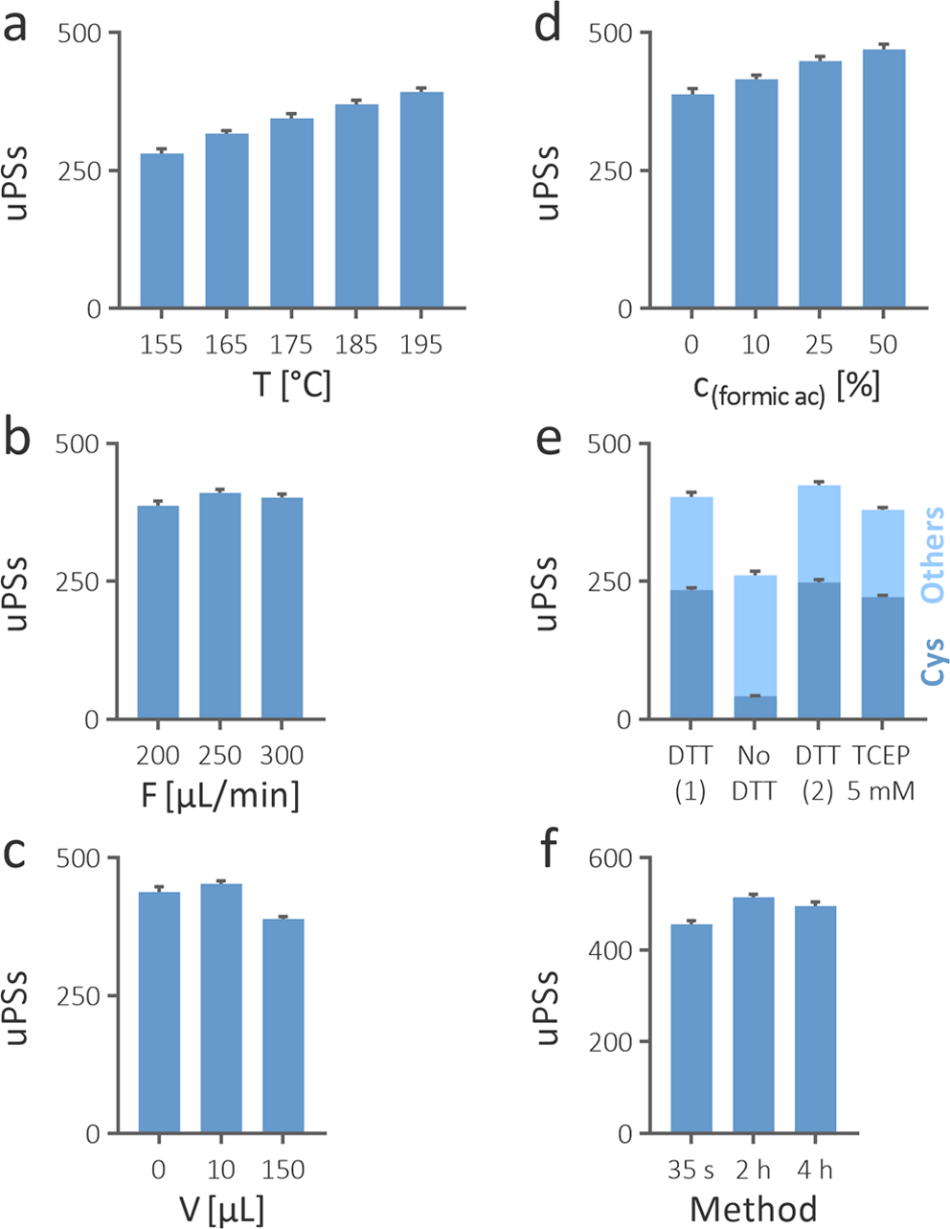
Effect of temperature of the reaction capillary (a), flow rate (b), gradient mixer volume (c), and formic acid concentration in the sample (d) on the number of identified unique peptide sequences (uPSs). The effect of reduction of disulfide bonds on uPSs and the portion of those containing Cys (e). Trastuzumab reduced in dithiothreitol (DTT) was prepared and analyzed separately for the non-reduced sample (no DTT) and the sample reduced in 5 mM tris(2-carboxyethyl)phosphine (TCEP 5 mM). Identified uPSs using our method (35 s) and the offline method in 2% formic acid at 108 °C for 2 and 4 h (f).

In line with the diminishing differences between the TIC and BP chromatograms and the positive trend in identified spectra, more uPSs were identified at higher temperatures (Figure 4a). Based on the number of uPSs, a temperature of 195 °C was the achievable optimum in our study and used in the next experiments. These results proved that the mobile phase containing a mere 0.1% formic acid acts as a surprisingly effective medium for acid cleavage of peptide bonds despite its pH at 195 °C being predicted to be 3.04 compared to 2.68 at room temperature.^55^

The acid cleavage dominantly occurred at the C-terminus of the Asp residue, as described elsewhere.^19−22^ Although its relative abundance in trastuzumab is only 4.2%, 45.0% of uPSs identified at 155 °C were terminated with Asp (Figure S4). However, the relative abundance of sequences terminated by Asp gradually decreased along with the increased temperature and was only 23.2% at 195 °C. These results suggested that trastuzumab needed to be cleaved also at other sites to obtain the optimum bottom-up data. Of notice is, for instance, the increase in the relative abundance of peptides terminated by Glu or Lys. We also inspected amino acids at N-terminus. Here, the relative abundance of sequences starting with Pro and Tyr showed a positive trend with increased reaction temperature. Nevertheless, deciphering the cleavage specificity using a single protein is tricky, and experiments with whole-proteome samples are necessary to characterize the complexity of the cleavage thoroughly.

We recently revealed a correlation between the temperature of the acidic mobile phase and of dehydration of Asp.^15^ The resulting succinimide impedes cleavage at Asp residue,^56^ thus representing a significant source of missed cleavages (Figure S5). Previous studies concerning acid cleavage of proteins often overlooked this unavoidable modification. Here, we confirmed its positive association with temperature with a plateau at 185 °C. Compared to Asp dehydration, oxidation of Met, which represents the most common artificial modification in bottom-up proteomics, was detected at a much lower level (Figure S6).

The combined sequence coverage of trastuzumab higher than 99.5% was obtained already in experiments carried out at 155 °C. At higher temperatures, the coverage was 100% for both chains, but at 185 and 195 °C, the C-terminal Lys of the heavy chain was not covered in all replicates. Overall, the sequence coverage was not informative and was not further evaluated. It should be noted that although we found 195 °C as the optimum temperature, more uPSs can be identified at different temperatures for other instruments. For instance, mass spectrometers equipped with electron-based dissociations may provide better results from larger, more charged peptides likely generated at lower temperatures.^57^

#### Flow Rate for Loading

We also expected that the time the sample spent in the reaction capillary could affect the efficiency of the cleavage. No metal capillary with an outer diameter of 1/16 in. and a length of 100 cm with a greater volume was found. Hence, we decreased the flow rate for delivering the sample in the column by 50 and 100 μL/min to tune the reaction time. Approximately 5 cm of both capillary ends protruded from the dry bath. The remaining 90 cm of the capillary was available for the acid cleavage, corresponding to approximate reaction times of 53, 42, and 35 s at the flow rates of 200, 250, and 300 μL/min, respectively.

No profound differences were observed among tested flow rates (Figure 4b). The most uPSs were identified at 250 μL/min. However, a 1.9% increase compared to the 300 μL/min was statistically insignificant. For this reason and because we preferred to keep the flow rate within the LC-MS method constant, we applied 300 μL/min in the following experiments. The loading flow rate had only a marginal effect on the cleavage specificity (Figure S7). The data confirmed that efficient acid cleavage could be achieved within 35 s. Such a short time is appealing for online integrating with LC-MS for fast protein analyses.

#### Volume of the Mixer

The sample used for the method development did not contain any acid. Therefore, the original configuration involved a 10 μL gradient mixer installed at the inlet of the reaction capillary to ensure mixing of the sample with the acidified mobile phase. To confirm its functionality, we replaced the mixer with a zero-dead-volume (ZDV) union. The 10 μL mixer yielded 3.1% more identified peptides than the ZDV union, but the difference was statistically insignificant. Hence, we also probed a 150 μL mixer to test whether the positive trend between identified uPSs and the mixing volume could lead to a statistically significant increase. Surprisingly, the larger mixer yielded 16.2% fewer identifications (Figure 4c). The explanation for these findings was elusive at this stage of the study. However, we later revealed that even the 10 μL mixer was an important source of carry-over (see the Carry-Over section). At that moment, we retrospectively hypothesized that the decrease in identifications was due to nonspecific protein adsorption in the large gradient mixer. It is worth noting in this context that gradient mixers are not components designed to come in contact with analytes. Because the 10 μL mixer provided no worse results than the ZDV union and worked as a union per se, we kept it in the configuration for the experiments until it was eventually removed. Globally, the mixer volume had no significant effect on the cleavage specificity (Figure S8).

#### Composition of the Sample Solvent

Our experiments confirmed that the acidification of the sample could be achieved by dispersing the injected band into the acidified mobile phase. The original procedures for offline acid cleavage of proteins were carried out using a 20-times higher concentration of formic acid.^21,22,24^ Hence, we were interested in whether the online cleavage could be potentiated by adding formic acid to the sample solvent. In this test, we also considered variable formylation at the N-terminus of peptides and Lys, Ser, and Thr residues.

ANOVA followed by Dunnett’s multiple comparisons test revealed that concentrations of formic acid of 10, 25, and 50% provided statistically more uPSs than the control sample (Figure 4d). At its highest concentration, 21% more uPSs were identified versus the control sample, confirming the positive effect of formic acid on cleavage efficiency.

Formic acid is known to induce formylation of proteins and peptides.^58−62^ Therefore, we diluted the samples for each concentration of formic acid just before the injection. Although the first injection of each sample was completed within 90 s after dilution, we found a substantial formylation. Its rate correlated with the concentration of formic acid in the sample (Figure S9). We did not observe a trend between the formylation and the injection order, indicating a fast reaction rate. A mere 0.4–0.6% of uPSs were identified exclusively using formylated peptides. Thus, formylation did not increase a chance of a peptide being identified. The higher content of formic acid in the sample did not activate other cleavage sites, and quite unexpectedly, the highest preference toward Asp-X cleavage was observed in the nonacidified sample (Figure S10). The increase in identified uPSs at higher concentrations was due to more efficient cleavage, arguably because the increased concentration of formic acid compensated for its lower acidity at 195 °C.^55^ A few formylated peptides were also identified in the control sample. Its analysis using mobile phase acidified with 0.5% acetic acid confirmed that these identifications were false positive hits resulting from inherently improper search settings in Byonic.^58^

A modification that can occur at several sites in a peptide enormously enlarges the search space, particularly when nonspecific cleavage is involved, increasing the chance of false positive identifications. In addition, if a portion of a peptide is formylated, the abundance of the parent peptide decreases. The sequence coverage of trastuzumab was almost 100% without adding formic acid to the sample. Thus, additional peptides identified from the acidified samples could not improve it anymore. Besides, we intended to use the final method for a quantitative analysis where the signal intensity mattered, and we wanted to avoid decreasing it due to the formylation of parent peptides. Therefore, we decided against using formic acid in the sample for the next experiments. However, we do not rule out that its positive effects could overrule its negative effects in some niche applications.

The Fenselau group used 12.5% acetic acid for acid cleavage of proteins accelerated by microwaves.^27,63^ Adding acetic acid to the sample solvent impaired the efficiency of our method. The number of uPSs decreased by 6% when trastuzumab was dissolved in 12.5 and 25% acetic acid, and a nonsignificant decrease of 1% was observed when the sample solvent contained even 50% acetic acid.

#### Reduction of Disulfide Bonds

Basile et al.^64^ suggested that high temperatures can break disulfide bonds in solid proteins. To test whether this can be achieved in solution at 195 °C, we compared the number of uPSs identified from reduced trastuzumab with those obtained from its nonreduced form. The analysis of the latter provided significantly fewer identifications (Figure 4e). The difference was associated with a drastic drop in sequences containing Cys, indicating that disulfide bonds were not reduced. These results corroborated earlier observations that the reduction is necessary for optimum acid cleavage.^28^

The reduction of disulfide bonds takes 0.5–1 h in typical protocols. Such time to prepare a ready-to-inject sample would disqualify our method from fast applications. TCEP is more effective than dithiothreitol and can break disulfide bonds at low pH.^65^ Hence, we tested whether TCEP can reduce trastuzumab while the dispersed mixture passes the reaction capillary. The reconstituted solution of trastuzumab was added to an HPLC vial containing 5 mM TCEP, immediately vortexed, and injected. This procedure almost restored the number of identified uPSs containing Cys (Figure 4e). The delay between mixing the sample with TCEP and its injection was around 90 s. A subsequent analysis of freshly diluted trastuzumab in 5 mM TCEP in the bypassed mode suggested that complete reduction was not achieved within that time. LC-UV analysis using a mobile phase acidified with 0.1% TFA for higher resolution confirmed that. We further simulated the conditions the sample underwent while passing the reaction capillary and placed an HPLC vial with fresh trastuzumab in 5 mM TCEP in the dry bath for 30 s. The LC-UV chromatograms indicated that the reduction of trastuzumab could be completed while passing the reaction capillary (Figure S11). The accelerated reduction did not represent any delay in the method and made it wholly suitable for fast protein analysis. Its implementation resulted in 11% fewer identified uPSs than from the injection of trastuzumab reduced with DTT for 30 min. Nevertheless, the sequence coverage remained the same.

All trastuzumab samples were kept in the presence of DTT or TCEP. We did not expect the free thiol groups to reoxidize in this environment and therefore did not block free thiols. This workflow spared significant time otherwise needed to block thiol groups quantitatively.^66,67^ Our decision was also motivated by a study demonstrating that maintaining the thiol residues free improved peptide identification in bottom-up analyses.^68^

#### Carry-Over

With the reaction capillary bypassed, the carry-over from the column was acceptable. However, significant signals were detected in a blank after trastuzumab injection when the reaction capillary was connected in the flow path. The 10 μL gradient mixer was identified as the major source of this carry-over. Only full-loop injections of formic acid washed out the adsorbed proteins efficiently. Because the gradient mixer was not critical for the efficiency (Figure 4c), it was replaced with a ZDV union. The carry-over became fully acceptable without the gradient mixer. After reduced trastuzumab was analyzed twice, 21 peptides were identified in the blank, and the number decreased to only 4 and 1 in subsequent injections.

#### Comparison to the Offline Procedure

**Figure 5:**
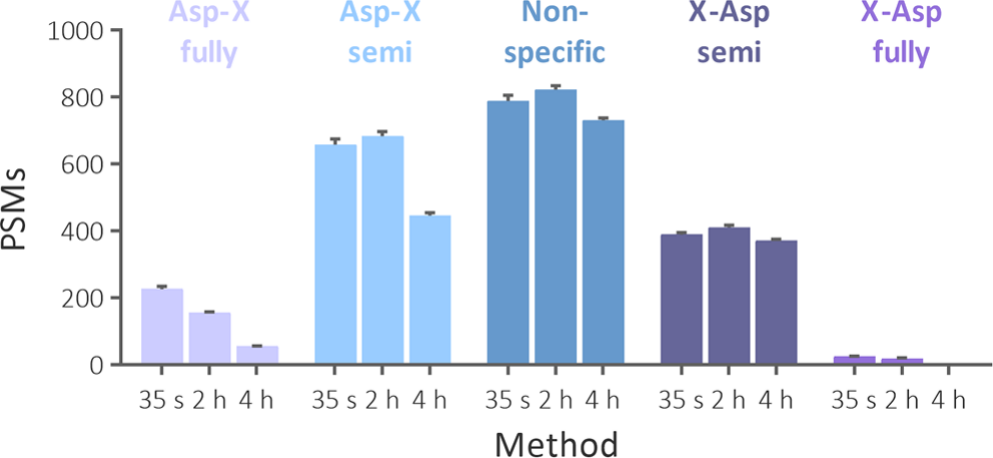
Number of peptide-spectrum matches (PSM) identified using various cleavage specificity from data obtained using our method (35 s) and the offline method for acid cleavage in 2% formic acid at 108 °C for 2 (2 h) and 4 h (4 h).

In the original procedure for the cleavage in formic acid, proteins were offline incubated in a 2% acid solution at 108 °C for 2 and 4 h.^21,22^ Our method involved markedly different concentrations of formic acid (0.1%), temperature (195 °C), and time (35 s). Therefore, we examined whether differences in results under such distinct conditions could be observed (Note S2). Despite the markedly different LC-MS chromatograms (Figure S12), the number of uPSs identified using a nonspecific search spanned within ±10% of the mean of all three conditions (Figure 4f). Our method resulted in 11.4% fewer uPSs versus those yielded from trastuzumab cleaved in 2% formic acid for 2 h. The offline method was reported to be very specific for Asp.^21,22^ Therefore, we tested whether the number of identified uPSs was larger because the cleavage in 2% formic acid generated principally specific peptides. Strikingly, the offline method generated more lower-specificity peptides (Figure 5). An unbiased investigation of both termini of identified uPSs did not reveal significantly different cleavage patterns (Figure S13). The data suggest that a high specificity is unachievable in the acid cleavage of proteins. We suppose this is very likely true also for methods derived from the original protocol and speculate that the earlier highlighted specificity toward Asp resulted from the absence of an unbiased investigation of the cleavage sites. The Venn diagram indicated that each method has certain selectivity, but most of the uPSs identified by our method were shared with a version of the offline procedure (Figure S14).

The rate of Asp dehydration was lower in the offline method, indicating that temperature is a more important factor than pH or time in this reaction. Nevertheless, Asp dehydration should also be considered for identifying spectra obtained using the offline method. The offline method used 2% formic acid. Hence, it was unsurprising that a significant portion of peptides was formylated. A significant level of Met oxidation was found in peptides generated by the offline method compared to ours (Figure S15). The drop at 4 h remained unexplained but was not due to additional oxidation to Met-sulfone.

Our accelerated online method does not seem to produce data more challenging to evaluate than the offline one, and in some qualitative aspects, data obtained by our method are superior.

#### Qualitative Repeatability

**Figure 6:**
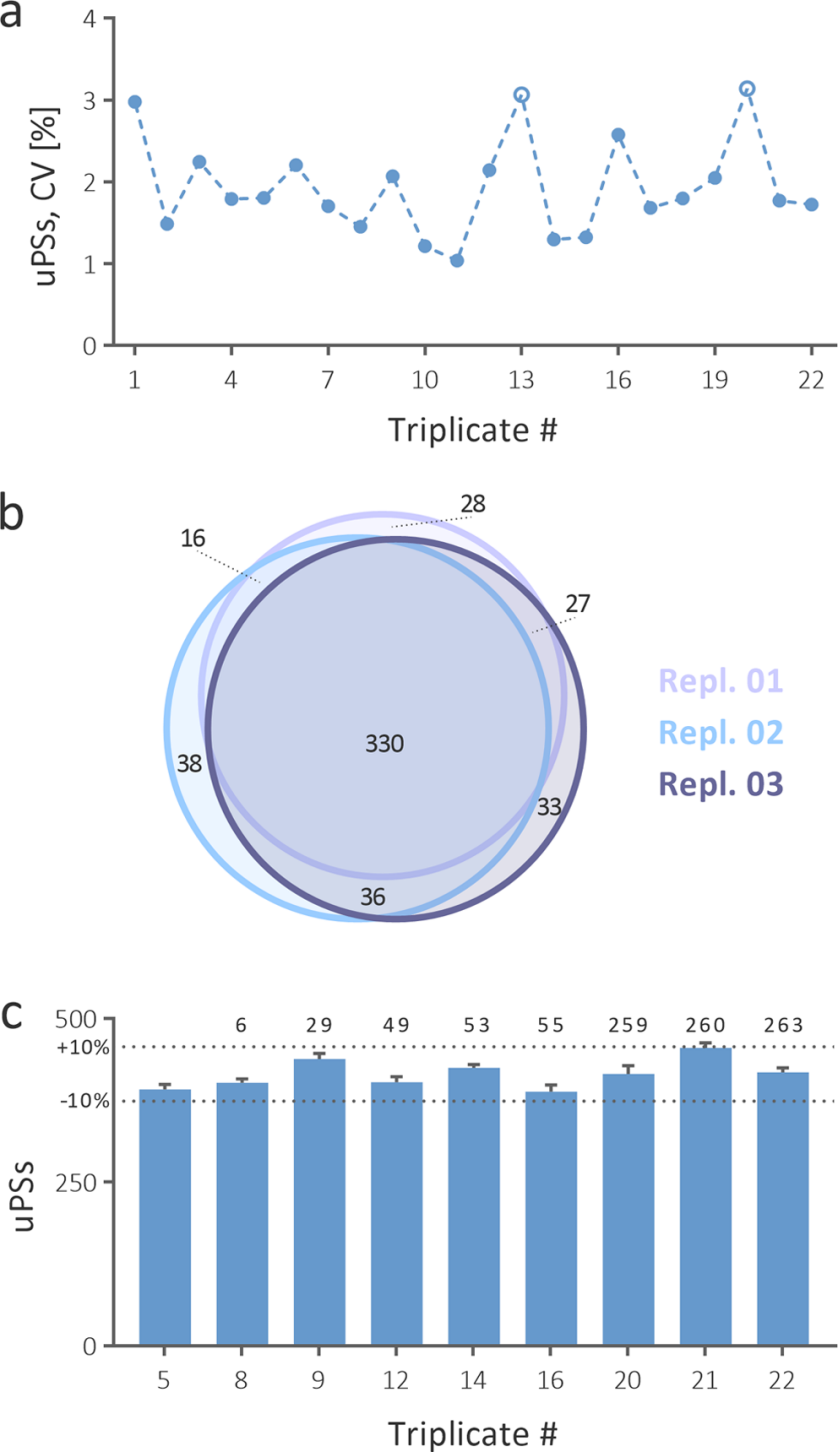
Run-to-run repeatability of our method in terms of the number of identified unique peptide sequences (uPSs) across 22 triplicates (a). Two triplicates with a CV greater than 3% are shown as circles. A quantitative Venn diagram showing unique peptide sequences identified repeatedly within triplicate #20 (b). Long-term repeatability of the method in terms of the number of identified uPSs across 9 triplicates measured under identical conditions (c). The time in days, since the first triplicate was analyzed is shown above each subsequent triplicate.

All optimization experiments with trastuzumab were done in triplicate. Regardless of the particular conditions, the highest coefficient of variation (CV) of identified uPSs was 3.1%. Except for one additional case, CV was below 3% in all remaining triplicates (Figure 6a). These statistics demonstrate very good overall run-to-run repeatability, considering that the observed variance combines the variance of the online acid cleavage and the variance in the performance of the LC-MS instrumentation. We hypothesize that the outstanding repeatability is at least in part due to the minimized manual handling of the sample. A Venn diagram constructed from data obtained from triplicate #20 with the highest CV shows that 65.0% of all uPSs identified in the triplicate were identified in all replicates (Figure 6b). Considering the stochasticity of data-dependent acquisition and the length of the effective elution window of the gradient, the chance of a peptide being repeatedly identified was very reasonable.

Identical experimental conditions were used in 9 triplicates. The time gap between triplicate #5 and triplicate #22 was 263 days. Despite such a long break, the number of identified uPSs spanned within ±10% of the mean (Figure 6c). Thus, our method also exhibits outstanding long-term repeatability, considering the numerous factors that potentially affect the performance of LC-MS over such a period of time.

#### Quantitative Repeatability

**Figure 7:**
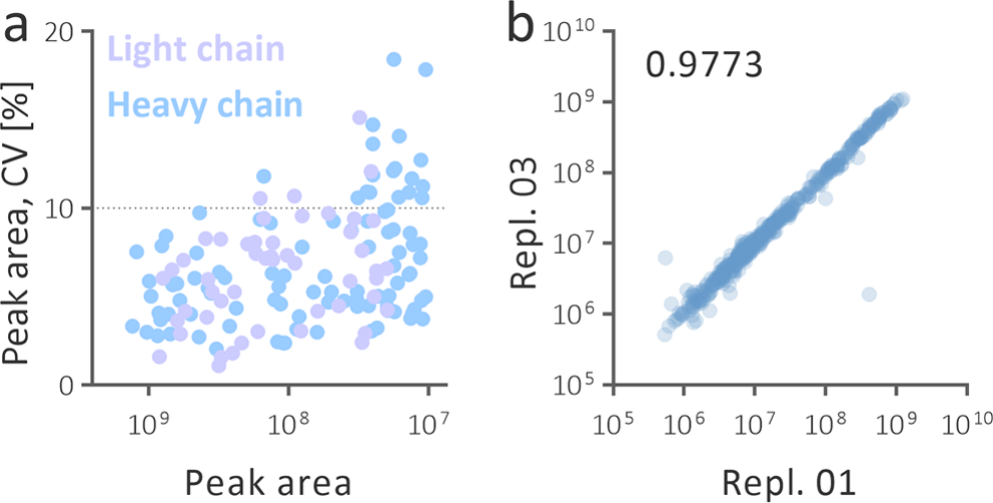
Quantitative repeatability of the most responsive peptides generated via the online acid cleavage (a). The coefficient of variation (CV) for 50 and 100 peptides from light and heavy chains of trastuzumab was calculated from six replicates. Correlation of LC-MS peak areas between replicate 1 and 3 with the worst observed Spearman coefficient (b).

Six consecutive analyses were carried out to examine the quantitative characteristics of the method. Quantifying proteins in bottom-up experiments is performed using the most intense peptides that typically suffer from the least variation in MS signal. Hence, we first evaluated the LC-MS peak area for 50 and 100 most responsive peptides from trastuzumab light and heavy chains. The peak area of those peptides spanned two orders of magnitude. The median CV of the peak area was 6.0% and did not cross 20% for any peptide. Only 14.7% of values were greater than 10% (Figure 7a). We concluded that the method could generate enough peptides for reliable quantification, even from small and moderate proteins.

The superb quantitative performance of the best responsive peptides motivated us to examine run-to-run quantitative repeatability regardless of the peak area of generated peptides. Skyline extracted the peak area for 432 precursors covering 324 uPSs in all six replicates. The worst Spearman coefficient in the individual correlations was 0.9773 (Figures 7b, S16), indicating that the method can provide a reliable MS signal over three orders of magnitude in a nontargeted mode.

#### Quantitative Response

After optimization of the method, we assessed its potential for protein quantification. Two microliters from trastuzumab solution with concentrations of 1 ng/μL to 1 μg/μL prepared in a half-log serial dilution were injected three times. Using Skyline software, we selected 15 peptides with the largest peak area. The most intense precursor for each peptide was kept. Quantitative data were evaluated for 12 peptides with LC-MS peaks detected across all dilutions. All those peptides resulted from cleavage at Asp residues.

Relative intensities of peptides in LC-MS chromatograms varied along the nominal concentration of trastuzumab, indicating a nonlinear behavior of the quantitative response. Even a regression using a quadratic curve with 1/*x*^2^ weighting resulted in large relative errors. After the log_10_ transformation, the data fitted cubic regression with very good coefficients of determination and very decent relative errors (Figure S17). The data indicated that our method could accurately quantify proteins even without internal standards.

The complex nonlinear nature of the quantitative response is very likely a result of a combination of two factors. The equilibrium concentration of the monitored peptides is unattainable as they are generated from longer precursor polypeptides and, at the same time, hydrolyzed to shorter ones. Besides, the individual hydrolytic reactions have concentration-dependent kinetics, with different rate constants for the hydrolysis of a peptide bond joining particular pairs of amino acids.^19,69^

### Application of the Method for Fast Ricin Detection

Our method can quickly provide information about a protein identity and involves minimal sample manipulation. The only necessary manual step represents mixing the sample with the TCEP solution that can be prepared in an HPLC vial. These characteristics are extraordinarily attractive for detecting toxic proteins. Therefore, we were interested in how the method would perform for the fast detection of ricin.

**Figure 8:**
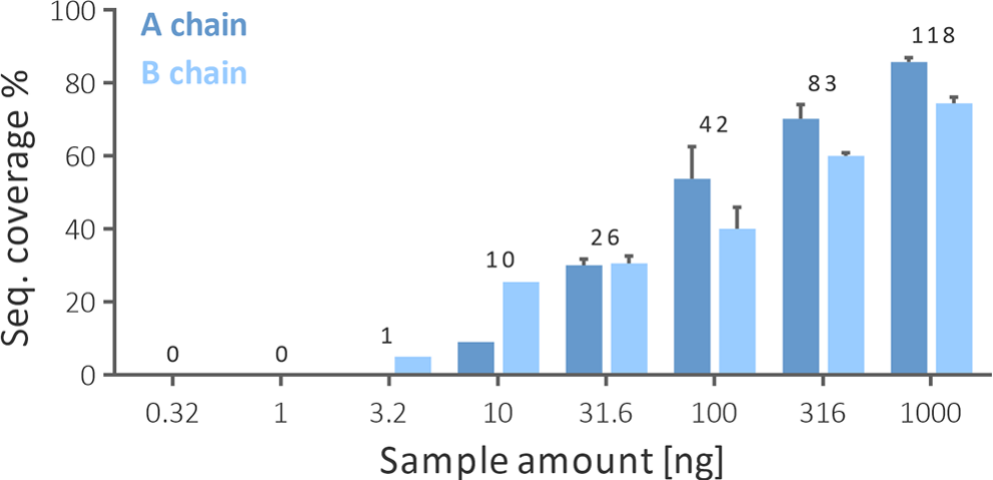
Sequence coverage of ricin chains and the average number of total identified peptides obtained from various injected sample amounts.

To make the whole procedure even faster, we excluded the 1 min isocratic step and shortened the gradient time to 3 min. The gradient span was reduced to 1–46% component B to utilize the gradient time most effectively. The total method time, including the column equilibration, was 9 min. We injected 2 μL three times from the ricin samples with a concentration of 0.16 to 500 ng/μL prepared in a half-log serial dilution. The minimum injected amount that provided identification of at least one peptide from each ricin chain in all three replicates was 10 ng (Figure 8). With the increased amount of ricin injected, the sequence coverage increased but did not reach 100%.

**Figure 9:**
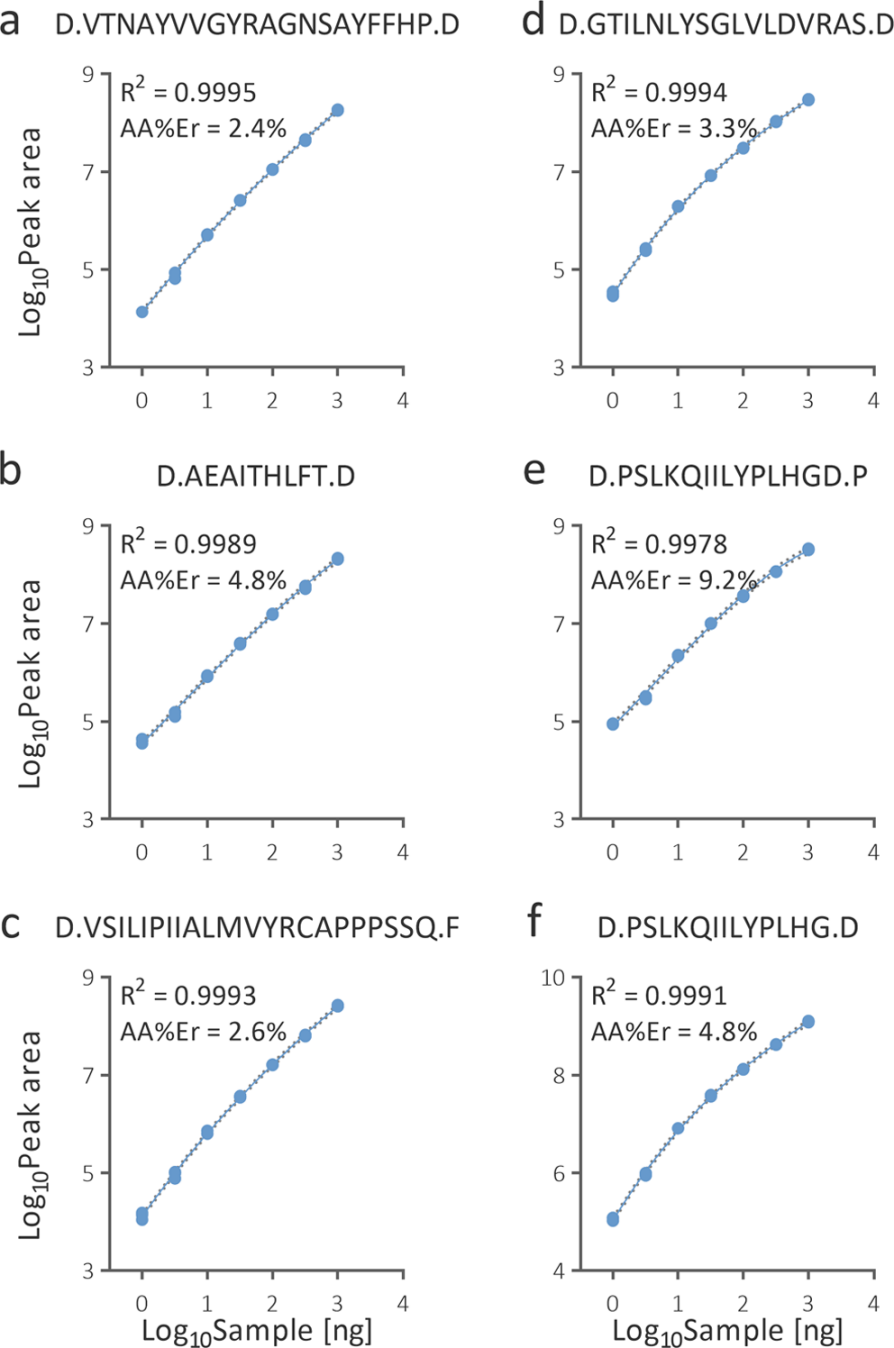
Quantitative performance of the three most intense peptides generated from ricin chain A (a–c) and chain B (d–f). Peptides AEAITHLFT and VSILIPIIALMVYRCAPPPSSQ are shared with castor bean agglutinin. Each replicate was considered as an individual point. Dot lines determine bands of 95% confidence.

The same LC-MS data were also used to construct calibration curves. The three best responsive peptides from each chain were used. Four peptides were specific for ricin, and sequences of two peptides were shared with castor bean agglutinin. Only one C-terminus in the evaluated peptides did not correspond to cleavage at Asp. The best parameters of a curve fit were obtained again using a cubic function after the log_10_ transformation of the input data (Figure 9).

The lowest concentration still produced reproducible signals across all peptides (CV ≤15%) was 0.5 ng/μL. At the lowest tested concentration of 0.16 ng/μL, the greatest MS response yielded the peptide D.PSLKQIILYPLHG.D with a CV of the peak area of 10.8%. The peak area was 16.2-fold higher than in the blank plus three standard deviations, i.e., significantly above the value recommended for determining a limit of detection. The sensitivity of the method was thus sufficient to detect ricin in the least quantities that would threaten humans.

In a simulated emergency alert, we examined the speed of ricin identification and the accuracy of its quantification. We injected 2 μL of 50 ng/μL ricin sample and analyzed the resulting peptides with the LC-MS method truncated to 5 min by removing the column equilibration step. Byonic identified 29 and 26 peptides from chains A and B within 30 s. The concentration determined based on calibration curves of six ricin peptides yielded a highly acceptable 7.6% error. Based on these results, we concluded that our method could identify ricin within as little as 7–8 min after receiving a suspicious material, and in a case of a positive outcome, it can determine its concentration accurately. To the best of our knowledge, no LC-MS-based proteomic method with such capabilities has been published yet.

### Final Refinement of the Method

The laboratory sand used in the dry bath was very fine, creating a risk of contamination of the environment in the LC-MS laboratory. Hence, after confirming the outstanding performance of the method, we replaced the sand with alumina beads of a 1.5–2.0 mm diameter. No effect on the stability of the temperature was observed, and no difference in obtained uPSs was registered (Figure S18). The number of shared uPSs between two triplicates obtained using different media was very similar to that of two triplicates obtained using the same media, indicating the medium did not affect the nature of generated peptides (Figure S19).

Additional experiments further confirmed that 0.1% formic acid in the mobile phase could be replaced by 0.5% acetic acid without changing the performance of the acid cleavage in terms of the number of uPSs (*p* = 0.51).

### Robustness of the Method

A total of 1338 analyses were carried out using the apparatus and the chromatographic column since the inception of the study without a need to replace any component due to wear out or degradation. The method is extremely robust, arguably because of its very simple design and the use of long-proven components and reagents. We believe that the simplicity of the method and the wide availability of the components allow its seamless adoption in any laboratory with experience with protein analysis.

## Conclusions

In the presented work, we developed and characterized a novel, extremely simple chemical method for online acid cleavage of proteins. The method is based on a low-cost apparatus fully integrable in standard LC-MS instrumentation. No sophisticated add-ons are needed, and the method does not require other chemicals except those standardly used for LC-MS in proteomics. The main advantages of the method are its simplicity, low cost, efficiency, speed, and seamless integration with LC-MS. Because the samples can be prepared directly in an HPLC vial and no additional sample manipulation is needed, the optimized method is virtually lossless. Besides, only the portion of the sample that LC-MS completely utilizes is processed. After optimization, our method demonstrated its outstanding qualitative and quantitative performance. As a proof-of-concept, we applied the method for the fast detection of ricin. That application proved that the method could identify ricin within several minutes after receiving a sample. We successfully tested the method also for identifying insulin (Figure S20) as a representative of small proteins and bacteriorhodopsin (Figure S21) as a representative of hydrophobic transmembrane proteins. Moreover, a mixture of four molecular weight protein standards ranging from 15 to 600 kDa (Table S1) and human saliva (Table S2) was successfully analyzed using the method. We trust that after tailored adjustments, the method holds great potential to be applied in the emerging area of fast and ultrafast proteomic analyses.

## Supporting Information Available

Additional results and discussion (Note S1); experimental details (Note S2); scheme of the apparatus with components (Figure S1); scheme of the instrumental setup (Figure S2); LC-MS chromatograms acquired at different temperatures of the reaction capillary (Figure S3); effect of temperature on the cleavage specificity (Figure S4); three reactions that Asp in polypeptides can undergo at acidic pH and high temperatures (Figure S5); effect of temperature on the artificial modifications (Figure S6); effect of the loading flow rate on the cleavage specificity (Figure S7); effect of the mixer volume on the cleavage specificity (Figure S8); effect of formic acid concentration on peptide formylation (Figure S9); effect of formic acid concentration on the cleavage specificity (Figure S10); LC-UV chromatograms of trastuzumab prepared with and without reduction (Figure S11); LC-MS chromatograms acquired using different methods (Figure S12); cleavage specificity of different methods of acid cleavage (Figure S13); comparision of unique peptide sequences identified using different methods of acid cleavage (Figure S14); artificially modified peptides generated using different methods (Figure S15); correlation matrix of LC-MS peak areas obtained in 6 replicates (Figure S16); quantitative performance of trastuzumab peptides (Figure S17); effect of the dry bath medium on the method performance (Figure S18); comparision of unique peptide sequences identified using different dry bath medium (Figure S19); results from online acid cleavage of human insulin (Figure S20); results from online acid cleavage of bacteriorhodopsin (Figure S21); results from online acid cleavage of a low-complexity protein mixture (Table S1); and results from online acid cleavage of human saliva (Table S2) (PDF)
