# Supplementary material for: Easy, Robust, and Repeatable Online Acid Cleavage of Proteins in Mobile Phase for Fast Quantitative LC-MS Bottom-Up Protein Analysis—Application for Ricin Detection
Source: Anal Chem. 2023 Aug 11;95(33):12339–48. doi: 10.1021/acs.analchem.3c01772 (PMC10448442; doi:10.1021/acs.analchem.3c01772)
Supplement: Supplementary file 1 — ac3c01772_si_001.pdf [file ac3c01772_si_001.pdf]

Supporting Information

Easy, robust, and repeatable online acid cleavage of proteins in mobile phase for fast quantitative LC-MS bottom-up proteomics – application for ricin detection

Denis K. Naplekov,[a†] Siddharth Jadeja,[a] Alena Fučíková,[b] František Švec,[a] Hana Sklenářová,[a] and Juraj Lenčo[a*†]

[a] Department of Analytical Chemistry, Faculty of Pharmacy, Charles University in Prague, Heyrovského 1203/8, 500 05 Hradec Králové, Czech Republic

[b] Biomedical Research Center, University Hospital Hradec Králové, Sokolská 581, 500 05 Hradec Králové, Czech Republic

*Corresponding Author: E-mail: lenco@faf.cuni.cz, Phone: +420 495 067 381.

† These authors contributed equally to this work

Table of Contents of Supporting Information

Note S1. Additional Results and Discussion 3
Method Development and Optimization 3
Note S2. Additional Experimental 4
Sample Preparation 4
Figure S1. Scheme of the apparatus used for the online acid cleavage of proteins in the mobile phase with labeled components 5
Figure S2. Scheme of the instrumental setup 6
Figure S3. LC-MS chromatograms acquired at different temperatures of the reaction capillary 7
Figure S4. Effect of the temperature on the cleavage specificity 8
Figure S5. Three reactions that Asp in polypeptides can undergo at acidic pH and high temperatures. 9
Figure S6. Effect of the temperature on the artificial modifications 10
Figure S7. Effect of the loading flow rate on the cleavage specificity 11
Figure S8. Effect of the mixer volume on the cleavage specificity 12
Figure S9. Effect of formic acid concentration on peptide formylation 13
Figure S10. Effect of formic acid concentration on the cleavage specificity 14
Figure S11. LC-UV chromatograms of trastuzumab prepared with and without reduction 15
Figure S12. LC-MS chromatograms acquired using different methods of acid cleavage 16
Figure S13. Cleavage specificity of different methods of acid cleavage 17
Figure S14. Comparision of unique peptide sequences identified using different methods 18
Figure S15. Artificially modified peptides generated using different methods 19
Figure S16. Correlation matrix of LC-MS peak areas obtained in 6 replicates 20
Figure S17. Quantitative performance of trastuzumab peptides 21
Figure S18. Effect of the dry bath medium on the method performance 22
Figure S19. Comparison of unique peptide sequences identified using different dry bath medium 23
Figure S20. Results from online acid cleavage of human insulin 24
Figure S21. Results from online acid cleavage of bacteriorhodopsin 25
Table S1. Results from online acid cleavage of a low-complexity protein mixture 26
Table S2. Results from online acid cleavage of human saliva 27
Additional References 28

Note S1. Additional Results and Discussion

Method Development and Optimization

Site for the online acid cleavage. Unsuccessful experiments with a 2.1 × 100 mm MAbPac RP column (Thermo Fisher Scientific) thermostated at 110 °C ruled out the possibility of efficient acid cleavage of proteins in a column. Therefore, we relocated the site for the acid cleavage to an upstream coiled metal capillary, allowing to use of higher temperatures while the cleavage products could still be separated. The slight drawback was a larger void volume, increasing the gradient delay by approximately 45 s at a flow rate of 300 µL/min.

Note S2. Additional Experimental

Sample Preparation

Offline acid cleavage of trastuzumab. The procedure introduced by Inglis and later adopted by Li et al. was used.[1,2] Briefly, 100 µL of reduced trastuzumab was mixed with 100 µL of 4% formic acid in an HPLC vial closed by caps with PTFE/silicone septa. Samples were incubated at 108 °C for 2 and 4 h in a column oven CTC 330 (SISw, Czech Republic). The injection volume for these samples was 4 µL corresponding to 2 µg trastuzumab. The reaction capillary was kept at room temperature during analyses of these samples.

Figure S1. Scheme of the apparatus used for the online acid cleavage of proteins in the mobile phase with labeled components

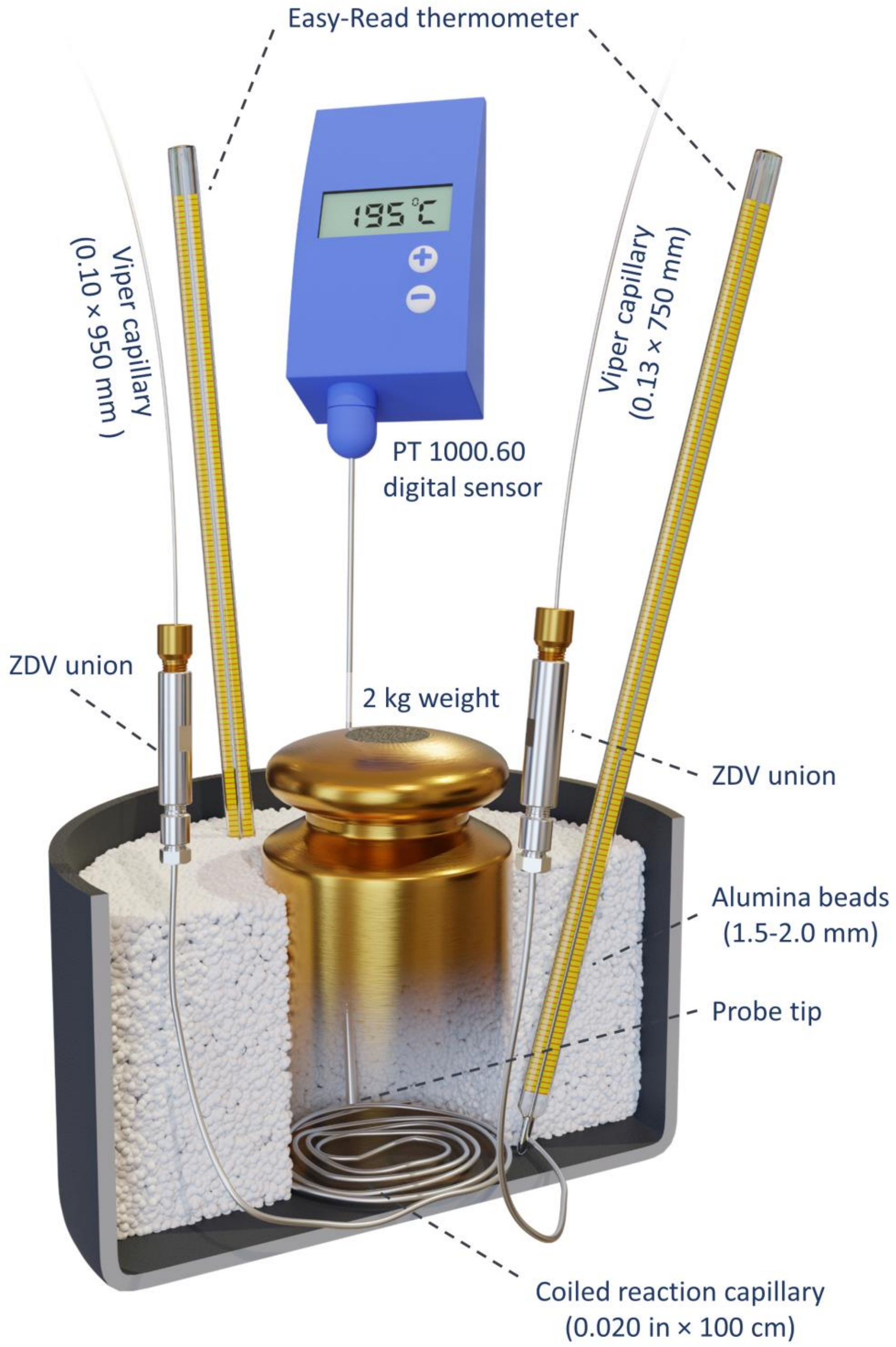

The final configuration is shown, i.e., without the gradient mixer and with the alumina beads.

Figure S2. Scheme of the instrumental setup

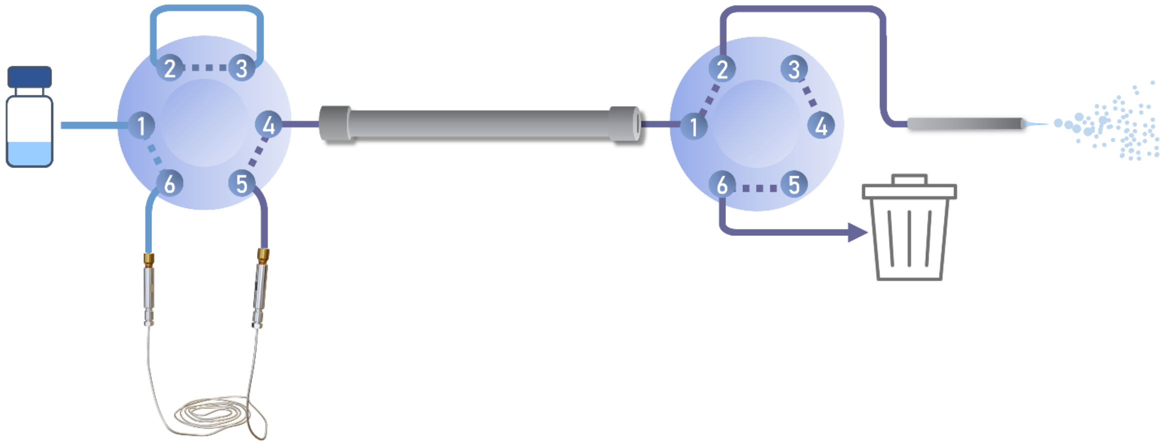

The reaction capillary was connected to the LC-MS system via a switching valve. The 10 µl gradient mixer (not shown here) installed at the inlet end of the reaction capillary was connected via a 0.13 × 750 mm Viper capillary (Thermo Fisher Scientific) to port 6 of a two-position six-port valve. The outlet of the reaction capillary was connected to port 5 via a 0.10 × 950 mm Viper capillary. Ports 2 and 3 were connected via a 0.10 × 150 mm Viper capillary so that the reaction capillary could be bypassed. The reaction capillary could have been connected directly between an autosampler and the column, but the valve allowed analyzing samples also in their uncleaved form. The column was connected to port 4 via a 0.10 × 380 mm Viper capillary with an active preheater. The inlet capillary from an autosampler was connected to port 1. Another switching valve was installed downstream of the column for diverting the injection peak to waste.

Figure S3. LC-MS chromatograms acquired at different temperatures of the reaction capillary

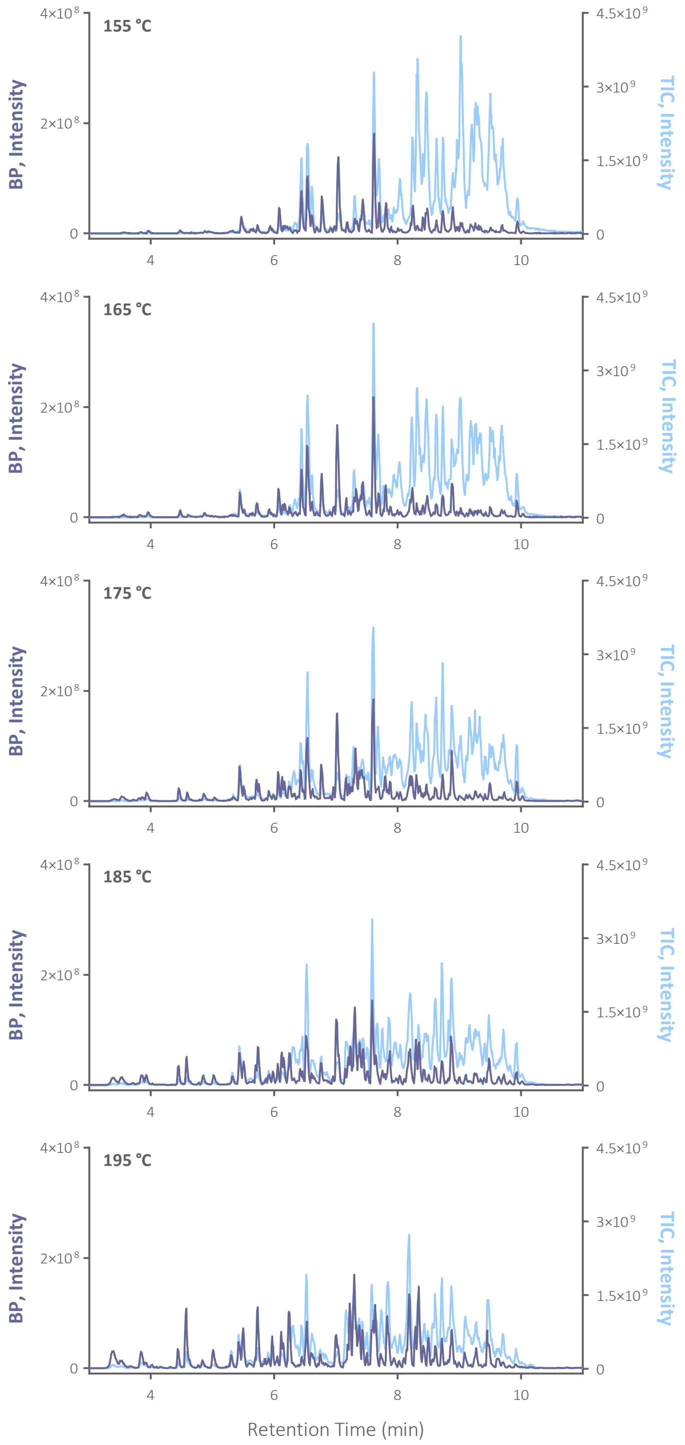

Total ion current (TIC) and base peak (BP) chromatograms acquired at temperatures of the reaction capillary between 155 °C and 195 °C. The chromatograms obtained at 195 °C are also presented in Figure 2.

Figure S4. Effect of the temperature on the cleavage specificity

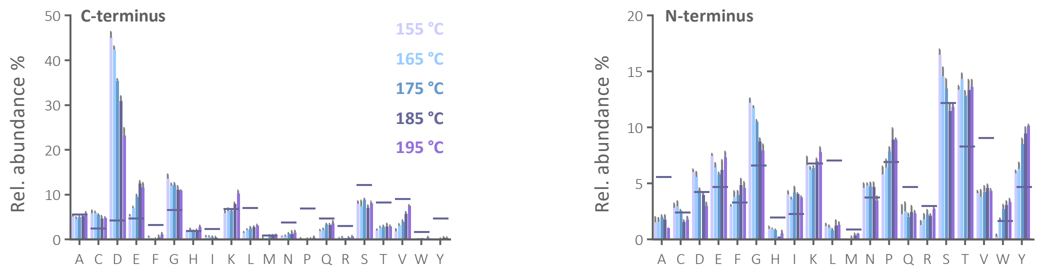

The effect of the temperature of reaction capillary on the relative abundance of amino acids at the C- and N-termini of unique peptide sequences. The horizontal lines above each residue represent their relative abundances in trastuzumab.

Figure S5. Three reactions that Asp in polypeptides can undergo at acidic pH and high temperatures.

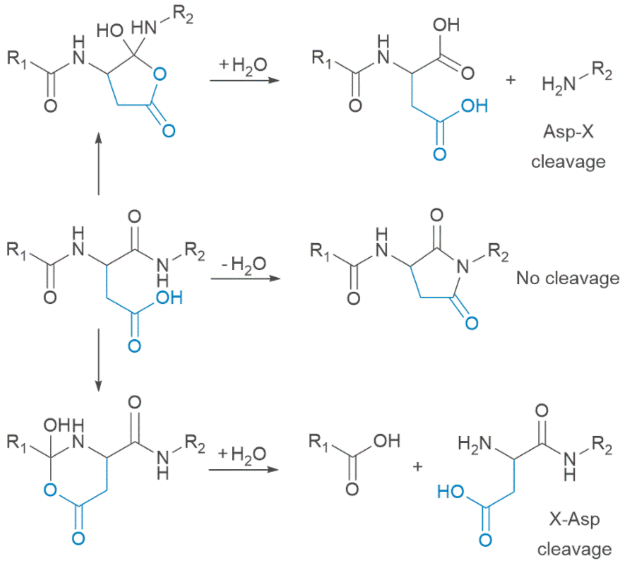

The dehydration of Asp results in succinimide, which prevents the hydrolysis of the peptide bond (middle). The other two reactions result in cleavage at the C-terminal (top) or N-terminal site (bottom) of Asp. The side chain of Asp is highlighted in blue.

Figure S6. Effect of the temperature on the artificial modifications

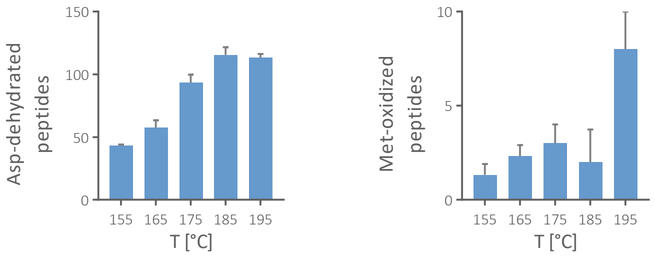

The trend between the temperature of the reaction capillary on the number of peptides with dehydrated Asp (left) and oxidized Met (right).

Figure S7. Effect of the loading flow rate on the cleavage specificity

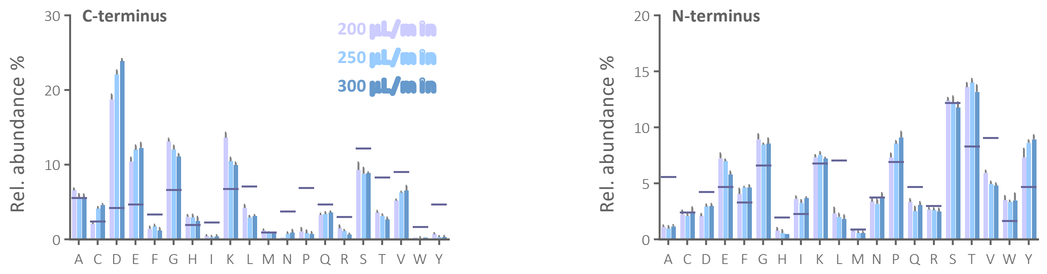

The effect of the loading flow rate on the relative abundance of amino acids at the C- and N-termini of unique peptide sequences. The horizontal lines above each residue represent their relative abundances in trastuzumab.

Figure S8. Effect of the mixer volume on the cleavage specificity

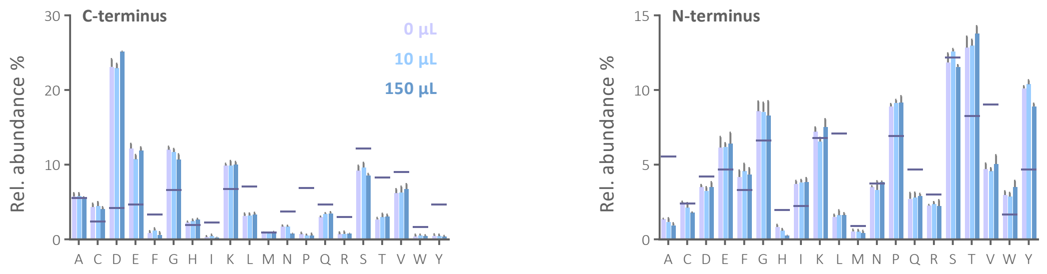

The effect of the mixer volume on the relative abundance of amino acids at the C- and N-termini of unique peptide sequences. The horizontal lines above each residue represent their relative abundances in trastuzumab.

Figure S9. Effect of formic acid concentration on peptide formylation

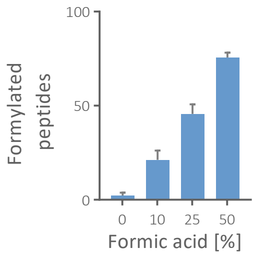

The effect of formic acid in the sample solvent on the number of identified formylated peptides.

Figure S10. Effect of formic acid concentration on the cleavage specificity

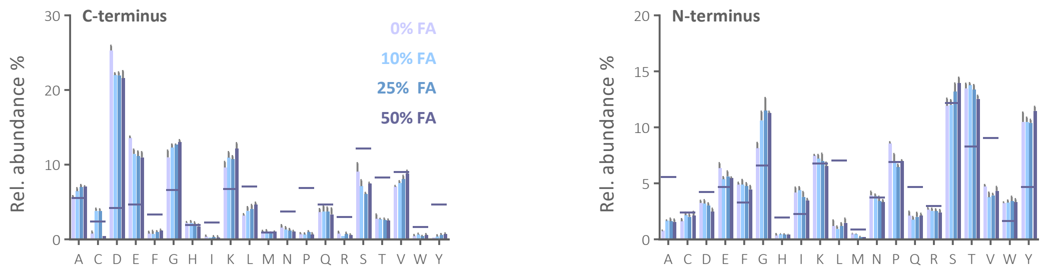

The effect of formic acid (FA) in the sample solvent on the relative abundance of amino acids at the C- and N-termini of identified unique peptide sequences. The horizontal lines above each residue represent their relative abundances in trastuzumab.

Figure S11. LC-UV chromatograms of trastuzumab prepared with and without reduction

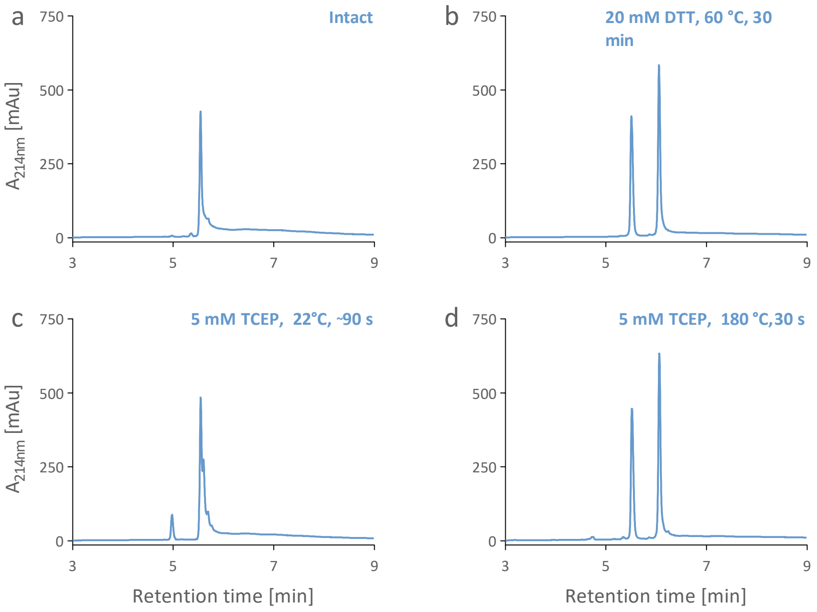

LC-UV chromatograms of trastuzumab samples prepared without reduction (a), with standard reduction using 20mM DTT (b), with reduction at room temperature in 5mM TCEP for ~90 s (c), and using an incubation in 5mM TCEP at 180 °C for 30 s (d). Very similar chromatograms were obtained from trastuzumab reduced in a standard way (b) and conditions mimicking the passage of the sample through the reaction capillary (d).

Figure S12. LC-MS chromatograms acquired using different methods of acid cleavage

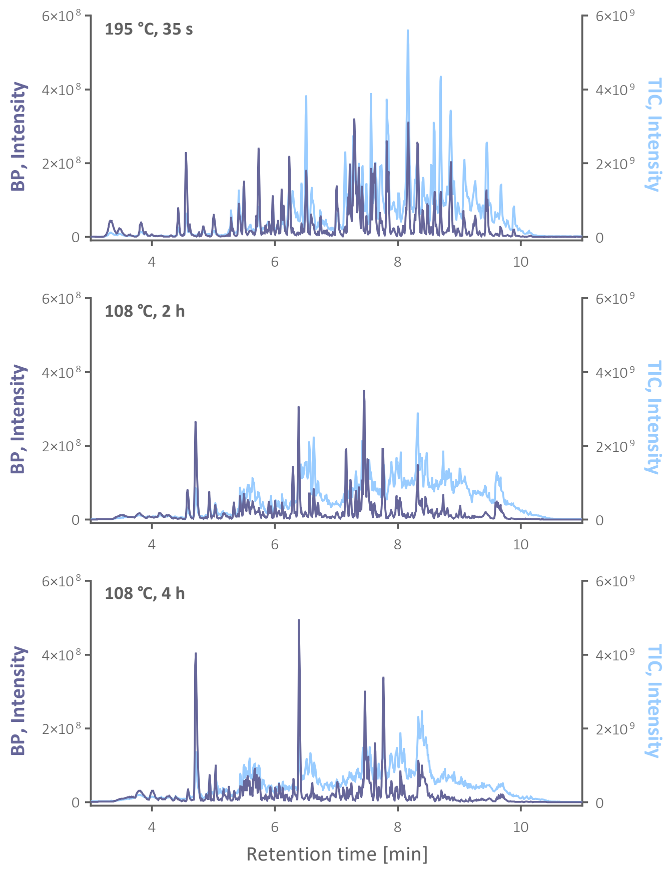

Total ion current (TIC) and base peak (BP) chromatograms acquired using our method (top chromatogram) and the offline method for acid cleavage in 2% formic acid at 108 °C for 2 and 4 hours (middle and bottom chromatograms).

Figure S13. Cleavage specificity of different methods of acid cleavage

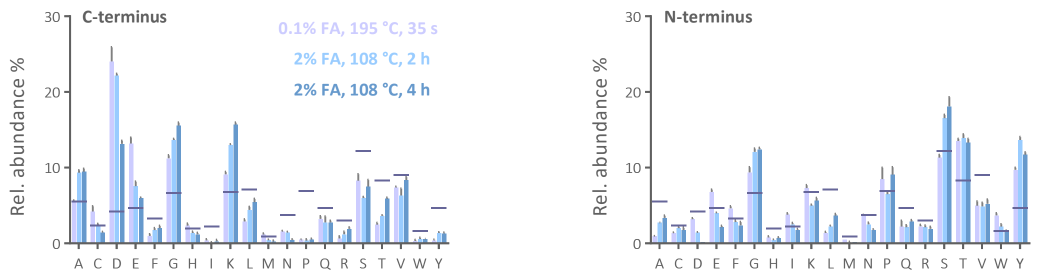

The comparison of our method with the offline procedure in terms of the relative abundance of amino acids in unique peptide sequences at their C-terminus (left) and N-terminus (right). The horizontal lines above each residue represent their relative abundances in trastuzumab.

Figure S14. Comparision of unique peptide sequences identified using different methods

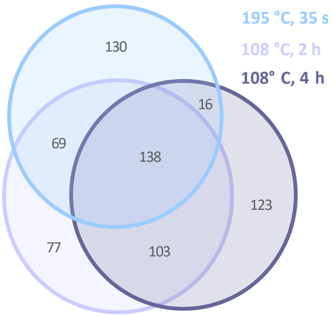

Differences in unique peptide sequences identified using our method (35 s) and the offline method for acid cleavage in 2% formic acid at 108 °C for 2 and 4 h.

Figure S15. Artificially modified peptides generated using different methods

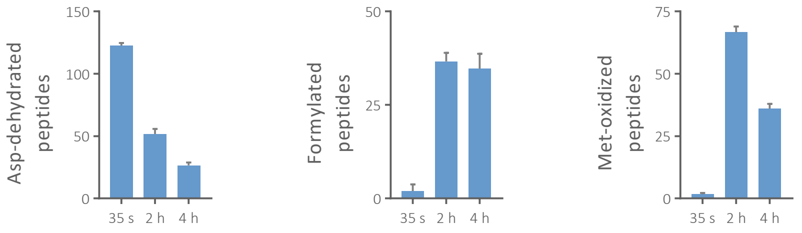

The number of peptides with dehydrated Asp, formylated peptides, and peptides with oxidized Met identified using our (35 s) and the offline methods (2 h and 4 h).

Figure S16. Correlation matrix of LC-MS peak areas obtained in 6 replicates

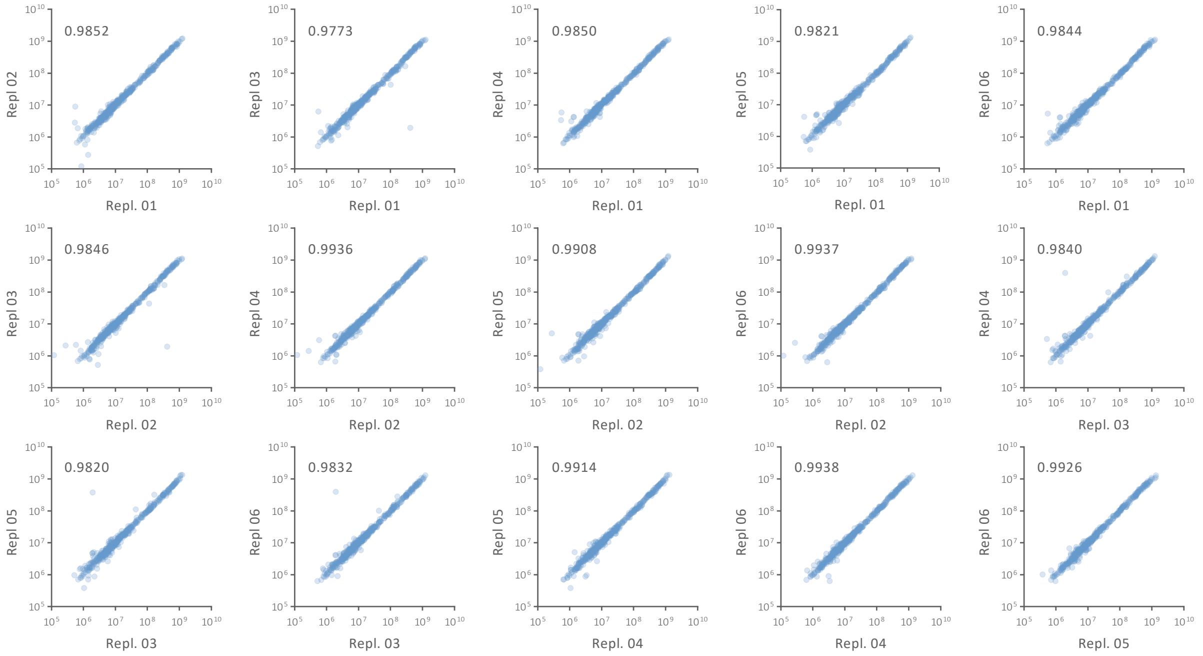

The correlation matrix of LC-MS peak areas extracted for 432 precursors of 324 unique peptide sequences across six replicates. The Spearman coefficient is shown for correlations of each replicate with each other replicate. The plot for the correlation of LC-MS peak areas between replicate 1 and 3 with the worst Spearman coefficient of 0.9773 is also presented in Figure 7b.

Figure S17. Quantitative performance of trastuzumab peptides

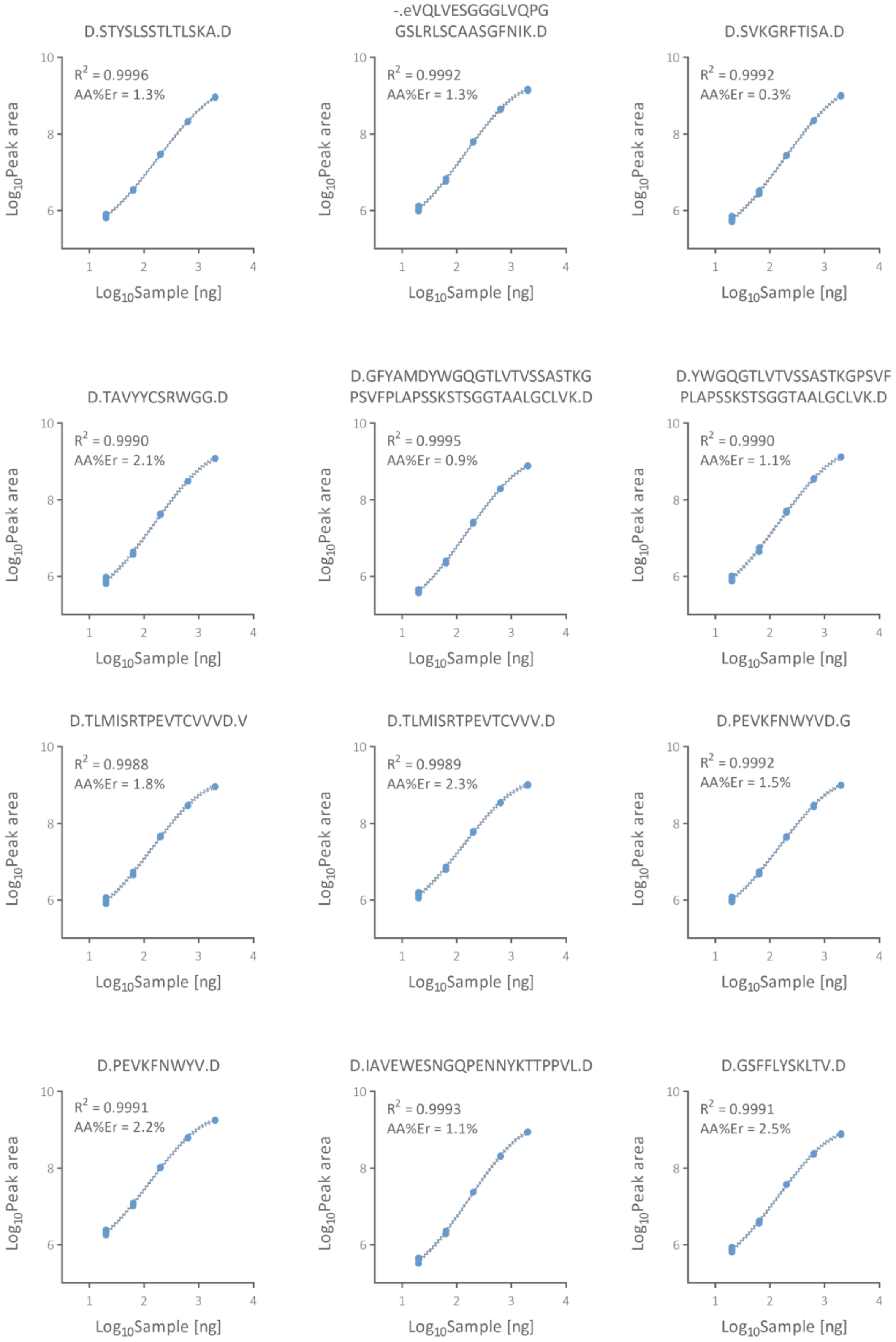

Quantitative performance of trastuzumab peptides evaluated using the coefficient of determination (R^2) and the average of the absolute values of the relative errors (AA%Er). Each replicate was considered as an individual point. Dot lines determine bands of 95% confidence.

Figure S18. Effect of the dry bath medium on the method performance

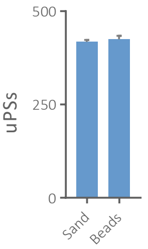

The effect of the dry bath medium on the number of identified unique peptide sequences (uPSs). Data obtained in six replicates are presented. Note: The incubation of trastuzumab in 5mM TCEP at 185 °C and all LC-UV analyses were already carried out with the alumina beads (Figure S8).

Figure S19. Comparison of unique peptide sequences identified using different dry bath medium

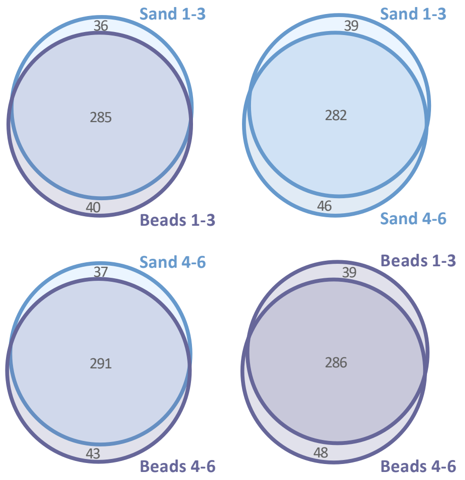

Unique peptide sequences shared between two triplicates obtained using the same or different dry bath media. Unique peptide sequences identified repeatedly in individual triplicates were evaluated. A total of 255 unique peptide sequences were shared among all four triplicates.

Figure S20. Results from online acid cleavage of human insulin

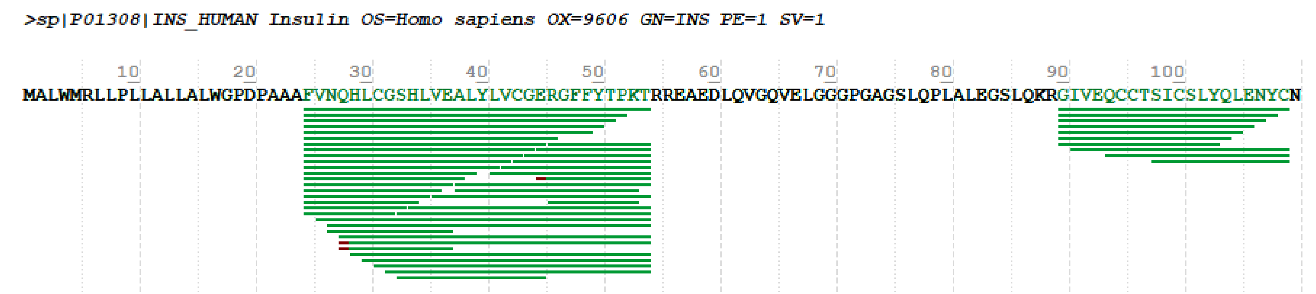

Insulin was prepared as described in the main text for trastuzumab. A volume of 2 µL containing 1 µg of reduced insulin was injected for the online acid cleavage in the mobile phase. The final method optimized using trastuzumab was used without any adjustments. Multiple acid cleavage events were induced, although the final insulin sequence does not contain Asp. FASTA sequence for preproinsulin was used for spectra identification. The final insulin molecule contains chains B (25-54) and chain A (90-110).

Figure S21. Results from online acid cleavage of bacteriorhodopsin

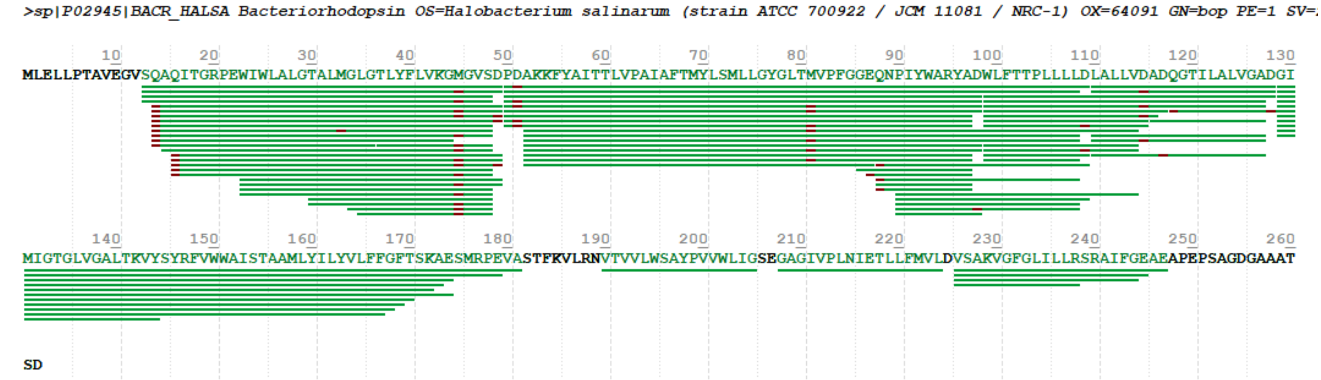

Bacteriorhodopsin from *H. salinarum* (HALOTEK Biotechnologie) was dissolved in 25% acetonitrile to a concentration of 1 µg/µL. A volume of 2 µL was injected for the online acid cleavage. The final method optimized using trastuzumab was used. The full FASTA sequence was used for spectra identification. The final bacteriorhodopsin molecule does not contain the propeptide (1-13).

Table S1. Results from online acid cleavage of a low-complexity protein mixture

Protein Rank	Description	|Log Prob|	Best |Log Prob|	Best score	# of spectra	# of unique peptides	# of mod peptides	Coverage %
1	>sp|P01012|OVAL_CHICK Ovalbumin OS=Gallus gallus	474.53	20.00	1226.60	121	95	27	84.97
2	>sp|P61823|RNAS1_BOVIN Ribonuclease pancreatic OS=Bos taurus	249.20	11.72	852.90	115	75	21	82.67
3	>sp|P01267|THYG_BOVIN Thyroglobulin OS=Bos taurus	215.03	9.67	707.90	52	51	4	26.22
4	>tr|A0A3Q1M3L6|A0A3Q1M3L6_BOVIN Ig-like domain-containing protein	159.13	9.95	775.00	33	32	2	54.77
5	>tr|G3N0V0|G3N0V0_BOVIN Ig-like domain-containing protein OS=Bos taurus	84.98	11.84	851.60	21	19	1	51.84
6	>sp|P01005|IOVO_CHICK Ovomucoid OS=Gallus gallus	73.12	13.18	950.10	22	21	5	56.19
7	>tr|F1N160|F1N160_BOVIN Ig-like domain-containing protein	45.16	6.59	646.20	13	12	0	39.43

The Protein Standard Mix 15 - 600 kDa for size exclusion chromatography was prepared as described in the main text for trastuzumab to a concentration of 2.5 µg/µL. A volume of 2 µL was injected for the online acid cleavage. The final method optimized using trastuzumab was used without any additional adjustments. The spectra were searched against a focused bovine and chicken FASTA database. Proteins identified using a minimum of 5 unique peptides are reported.

Table S2. Results from online acid cleavage of human saliva

Protein Rank	Description	|Log Prob|	Best |Log Prob|	Best score	# of spectra	# of unique peptides	# of mod peptides	Coverage %
1	>sp|P04745|AMY1_HUMAN Alpha-amylase 1	843.81	20.36	1201.60	232	168	32	88.26
2	>sp|P04280|PRP1_HUMAN Basic salivary proline-rich protein 1	732.12	29.95	1876.80	197	113	7	92.35
3	>sp|Q96DA0|ZG16B_HUMAN Zymogen granule protein 16 homolog B	508.86	22.09	1347.30	82	65	24	57.69
4	>sp|P01876|IGHA1_HUMAN Immunoglobulin heavy constant alpha 1	432.66	17.65	1100.20	64	58	4	77.90
5	>sp|P02814|SMR3B_HUMAN Submaxillary gland androgen-regulated protein 3B	366.44	13.77	867.90	93	74	25	72.15
6	>sp|P02810|PRPC_HUMAN Salivary acidic proline-rich phosphoprotein 1/2	326.75	21.29	1244.30	83	63	12	79.52
7	>sp|P10163|PRB4_HUMAN Basic salivary proline-rich protein 4	246.48	11.01	778.00	138	71	10	81.29
8	>sp|P23280|CAH6_HUMAN Carbonic anhydrase 6	234.71	23.13	1336.20	24	22	4	62.34
9	>sp|P01037|CYTN_HUMAN Cystatin-SN	183.51	14.27	936.60	40	33	14	84.40
10	>sp|P13646|K1C13_HUMAN Keratin, type I cytoskeletal 13	167.80	11.90	801.90	31	28	4	63.76
11	>sp|P19013|K2C4_HUMAN Keratin, type II cytoskeletal 4	150.01	12.67	881.50	33	29	3	46.15
12	>sp|P05109|S10A8_HUMAN Protein S100-A8	129.97	20.92	1295.80	13	13	0	91.40
13	>sp|Q96DR5|BPIA2_HUMAN BPI fold-containing family A member 2	107.47	14.48	878.10	20	14	2	55.42
14	>sp|P01833|PIGR_HUMAN Polymeric immunoglobulin receptor	106.27	13.94	875.10	27	19	3	34.16
15	>sp|P06702|S10A9_HUMAN Protein S100-A9	98.34	18.52	1183.00	17	12	5	83.33
16	>sp|P01834|IGKC_HUMAN Immunoglobulin kappa constant	94.88	21.03	1253.00	12	12	5	67.29
17	>sp|P12273|PIP_HUMAN Prolactin-inducible protein	89.31	11.29	783.00	43	23	7	76.71
18	>sp|Q04118|PRB3_HUMAN Basic salivary proline-rich protein 3	69.13	11.04	764.60	71	27	2	73.14
19	>sp|P02812|PRB2_HUMAN Basic salivary proline-rich protein 2	67.33	29.97	1889.60	16	16	2	67.31
20	>sp|P02768|ALBU_HUMAN Serum albumin	62.73	14.95	952.50	13	13	2	21.84
21	>sp|P61626|LYSC_HUMAN Lysozyme C	60.04	12.02	843.30	22	15	1	77.70
22	>sp|P28325|CYTD_HUMAN Cystatin-D	57.45	18.76	1120.70	9	8	6	67.61
23	>sp|Q9UBD6|RHCG_HUMAN Ammonium transporter Rh type C	54.27	23.25	1375.20	5	5	0	40.29
24	>sp|P63261|ACTG_HUMAN Actin, cytoplasmic 2	49.71	10.65	777.30	11	11	0	33.87
25	>sp|P48668|K2C6C_HUMAN Keratin, type II cytoskeletal 6C	46.67	11.53	759.20	14	11	2	24.82
26	>sp|P22079|PERL_HUMAN Lactoperoxidase	42.09	12.18	804.70	5	5	1	14.75
27	>sp|P25311|ZA2G_HUMAN Zinc-alpha-2-glycoprotein	41.14	8.03	615.80	11	10	0	38.93
28	>sp|Q6P5S2|LEG1H_HUMAN Protein LEG1 homolog	38.74	10.83	751.50	11	10	3	25.15
29	>sp|P01877|IGHA2_HUMAN Immunoglobulin heavy constant alpha 2	36.92	14.83	974.70	5	5	0	13.82
30	>sp|Q6MZM9|PRR27_HUMAN Proline-rich protein 27	31.56	11.20	774.40	4	4	0	35.62
31	>sp|P31025|LCN1_HUMAN Lipocalin-1	28.95	19.66	1186.40	3	3	0	48.86
32	>sp|P06703|S10A6_HUMAN Protein S100-A6	27.94	11.09	726.50	3	3	3	47.78
33	>sp|P04083|ANXA1_HUMAN Annexin A1	27.92	6.22	507.80	9	7	2	24.86
34	>sp|P02808|STAT_HUMAN Statherin	20.60	3.74	558.90	24	14	0	67.74
35	>sp|P01036|CYTS_HUMAN Cystatin-S	20.27	8.09	645.60	9	8	6	46.10
36	>sp|P15516|HIS3_HUMAN Histatin-3	17.80	5.23	520.20	15	7	0	47.06
37	>sp|Q8N4F0|BPIB2_HUMAN BPI fold-containing family B member 2	17.05	7.83	633.80	5	5	1	20.96
38	>sp|P01591|IGJ_HUMAN Immunoglobulin J chain	15.88	5.71	503.90	5	5	0	33.96
39	>sp|P06870|KLK1_HUMAN Kallikrein-1	15.36	5.97	594.60	3	3	0	21.37
40	>sp|Q8TAX7|MUC7_HUMAN Mucin-7	12.90	7.52	633.80	2	2	1	5.04
41	>sp|Q9HC84|MUC5B_HUMAN Mucin-5B	11.99	9.18	694.00	6	6	1	2.79
42	>sp|Q8NFU4|FDSCP_HUMAN Follicular dendritic cell secreted peptide	11.14	6.67	424.80	2	2	2	57.65
43	>sp|B9A064|IGLL5_HUMAN Immunoglobulin lambda-like polypeptide 5	10.01	3.58	449.50	5	5	1	17.76
44	>sp|P31949|S10AB_HUMAN Protein S100-A11	8.41	8.44	689.80	1	1	0	15.24
45	>sp|P15515|HIS1_HUMAN Histatin-1	7.64	3.48	406.30	4	4	1	26.32
46	>sp|P05164|PERM_HUMAN Myeloperoxidase	5.59	5.63	439.60	3	3	1	6.04
47	>sp|Q15485|FCN2_HUMAN Ficolin-2	5.54	2.50	389.90	5	4	0	10.54
48	>sp|Q8TDL5|BPIB1_HUMAN BPI fold-containing family B member 1	5.53	4.52	303.50	2	2	0	9.71
49	>sp|Q16378|PROL4_HUMAN Proline-rich protein 4	5.38	5.08	510.90	2	2	1	12.69
50	>sp|P24158|PRTN3_HUMAN Myeloblastin	4.71	4.68	563.00	2	2	0	7.03
51	>sp|A6NMY6|AXA2L_HUMAN Putative annexin A2-like protein	4.70	4.67	367.20	2	2	1	10.62
52	>sp|P09228|CYTT_HUMAN Cystatin-SA	4.65	4.70	351.50	1	1	1	13.48

A required volume of human saliva was added to accurately weighted dithiothreitol to create a 20 mM DTT solution. The sample was incubated at 60 °C for 30 min. A volume of 10 µL was injected for the online acid cleavage. The flow rate during the first three minutes after injection was decreased to 100 µL/min for better results. All other parameters were identical to those used in the final method optimized using trastuzumab. The spectra were searched against a focused human FASTA database. Proteins up to the occurrence of the first reverse protein identification are listed.

Additional References

(1) Inglis, A. S. In *Methods in Enzymology*, C.H.W. Hirs, S. N. T., Ed.; Academic Press, 1983, pp 324-332.
(2) Li, A.; Sowder, R. C.; Henderson, L. E.; Moore, S. P.; Garfinkel, D. J.; Fisher, R. J. *Anal Chem* **2001**, *73*, 5395-5402.
